# Design, Synthesis, and Evaluation of Acetylcholinesterase and Butyrylcholinesterase Dual-Target Inhibitors against Alzheimer’s Diseases

**DOI:** 10.3390/molecules25030489

**Published:** 2020-01-23

**Authors:** Yan Guo, Hongyu Yang, Zhongwei Huang, Sen Tian, Qihang Li, Chenxi Du, Tingkai Chen, Yang Liu, Haopeng Sun, Zongliang Liu

**Affiliations:** 1 School of Pharmacy, Key Laboratory of Molecular Pharmacology and Drug Evaluation (Yantai University), Ministry of Education, Collaborative Innovation Center of Advanced Drug Delivery System and Biotech Drugs in Universities of Shandong, Yantai University, Yantai 264005, China; 2 School of Pharmacy, China Pharmaceutical University, Nanjing 211198, China; 3 School of Traditional Chinese Pharmacy, China Pharmaceutical University, Nanjing 211198, China; 4 Jiangsu Food and Pharmaceutical Science College, Huaian 223003, China

**Keywords:** Alzheimer’s disease, acetylcholinesterase inhibitor, butyrylcholinesterase inhibitor, cytotoxicity, molecular docking, structural modification, structure-activity relationship

## Abstract

A series of novel compounds **6a**–**h**, **8i**–**1**, **10s**–**v**, and **16a**–**d** were synthesized and evaluated, together with the known analogs **11a**–**f**, for their inhibitory activities towards acetylcholinesterase (AChE) and butyrylcholinesterase (BChE). The inhibitory activities of AChE and BChE were evaluated in vitro by Ellman method. The results show that some compounds have good inhibitory activity against AChE and BChE. Among them, compound **8i** showed the strongest inhibitory effect on both AChE (eeAChE IC_50_ = 0.39 μM) and BChE (eqBChE IC_50_ = 0.28 μM). Enzyme inhibition kinetics and molecular modeling studies have shown that compound **8i** bind simultaneously to the peripheral anionic site (PAS) and the catalytic sites (CAS) of AChE and BChE. In addition, the cytotoxicity of compound **8i** is lower than that of Tacrine, indicating its potential safety as anti-Alzheimer’s disease (anti-AD) agents. In summary, these data suggest that compound **8i** is a promising multipotent agent for the treatment of AD.

## 1. Introduction

According to the report of the International Alzheimer’s Association in the last three years, there are more than 36 million AD patients in the world. With the acceleration of the aging process of the population, the amount of AD incidence is increasing year by year, and the number of AD patients in the world will exceed 130 million by 2050 [1,2,3]. The incidence of AD is over 1.9% among people over 60 years old in China [4]. AD is the third most common disease in the elderly after cardio-cerebrovascular disease and cancer. AD can cause dementia, which is one of the six leading causes of death in the United States [5,6].

AD was initially characterized by memory loss, cognitive dysfunction, inability to take care of themselves in daily life, and subsequent exacerbations of mental and behavioral abnormalities [7,8]. AD is not only a seriously threaten of human health and life, but also brings heavy mental burden and economic pressure to the family members and friends of the patients, causing huge fluctuations in the social economy. Therefore, it is an important task of medicinal chemists to develop effective drugs for the treatment of AD [9,10].

AD is a complex neurodegenerative syndrome. Due to the complicated pathogenesis of AD, its etiology is not completely clear. The therapeutic drugs used in the clinical and research stages can only delay the course of AD, but there are no drugs that cure or delay the course of AD [11,12]. A large number of studies have shown that hypotheses of the pathogenesis of AD include the theory of cholinergic damage, tau protein hyperphosphorylation, amyloid β-protein (Aβ) cascade hypothesis, metal ion homeostasis theory, APOE genotype, oxidative stress theory and so on [13,14,15,16,17,18].

Previous studies have identified three pathological features of AD: decreased levels of AChE in the neurotransmitter matrix, deposition of Aβ, and hyperphosphorylation of tau protein. Scientists have been trying to find the etiology and treatment strategy of AD through further study of the above three pathological characteristics [19,20]. At present, acetylcholinesterase inhibitors (AChEIs) are the main treatment for AD. Only five drugs have been approved by the Food and Drug Administration (FDA) to treat AD. Four of them are AChEIs, including Tacrine, Donepezil, Rivastigmine, and Galantamine. Tacrine is the first generation AChEI, but it is limited in clinical usage because of hepatotoxicity [11,21,22].

There are two types of ChEs in the central nervous system, namely AChE and BChE. AChE and BChE are important targets for the development of anti-AD drugs. The physiological function of AChE has been preliminarily understood, but the understanding of BChE is less. Studies have shown that ACh activity in some brain regions of patients with mild to severe AD decreases to 10%–15% of the normal value, and AChEIs have significant effects on patients with mild to moderate AD and can repair their cognitive impairment and other symptoms [23].

When AD develops to the middle and late stage, AChE activity decreases, whereas BChE activity increases, and BChE acts as a metabolic compensation for AChE, partially compensating for the role of AChE in hydrolyzing ACh [11,23,24]. The regulation of AChE is increasingly dependent on BChE, so BChE is gradually accepted as a target of anti-AD drugs. Therefore, the design and development of dual-target inhibitors of AChE and BChE may have the following advantages: it can not only effectively reduce the degradation of AChE and the drug resistance of AChEIs, but also be effective in patients for moderate to severe symptoms of AD. Some people think that appropriate inhibition of AChE and BChE is a more ideal treatment for AD [25,26]. Therefore, we are looking for dual-target ChEs inhibitors with inhibitory activity on both AChE and BChE.

With the study of AChE crystal structure, it was found that the ligand binding pocket of AChE in general is a long and narrow channel extending from the surface to the interior [27]. The channel is dumbbell-shaped. The opening and bottom of the channel are relatively open, and the middle is narrow. The active site of AChE contains two important domains: CAS at the bottom of the channel is the binding site for substrates and inhibitors, consisting of three residues: Ser203, His447, and Glu334; PAS is situated at the opening of the channel, which is the binding site for the enzyme inhibitor. It consists of five residues: Tyr72, Tyr124, Trp286, Tyr34 and Asp74 [11,28,29]. It was found that PAS of AChE could induce the formation of Aβ protein and accelerate its precipitation. Researchers began to develop dual-site AChEIs that act on both CAS and PAS sites simultaneously, which can interfere with the aggregation of Aβ while enhancing the inhibitory activity of AChE, and play a dual role in the treatment of AD [30].

In our previous studies, we found several new ChEIs through virtual screening based on pharmacophore. We have found that **G801-0274** AChE (eeAChE IC_50_ = 2.05 μM), BChE (eqBChE IC_50_ = 0.03 μM) can inhibit ChEs. In this paper, we used it as the lead compound for structural modification [31]. It has the property of dual-site binding and can bind PAS and CAS sites simultaneously.

**Figure 1:**
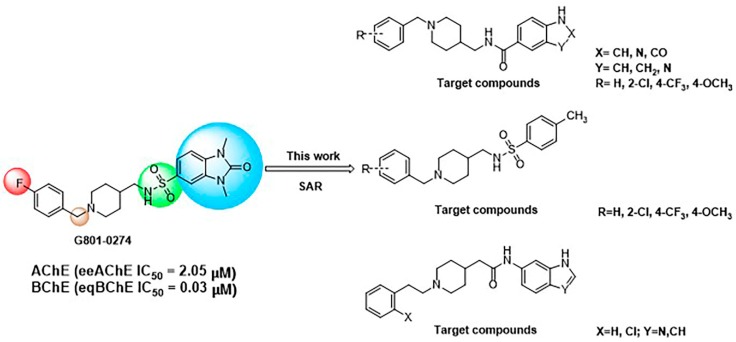
Design of a series of derivatives as dual-target inhibitor of AChE and BChE.

In this study, we designed and synthesized a series of new derivatives based on **G801-0274** and evaluated their biological activities, including cytotoxicity and AChEs inhibition. By summarizing our data, we found a new type of dual-target inhibitor of AChE and BChE with in vitro activities, hoping to develop anti-AD drugs through further efforts (Figure 1).

## 2. Results and Discussion

### 2.1. Chemistry

**Scheme 1:**
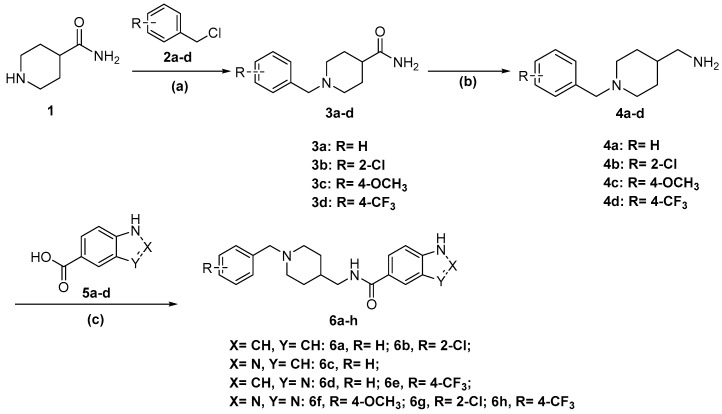
Synthesis of compounds **6a**–**h**. Reagents and conditions: (**a**) compounds **2a**–**d**, KI, K_2_CO_3_, acetone, reflux 4 h; (**b**) LiAlH_4_, THF, 0 °C, reflux 4 h; (**c**) **5a**–**d**, CDI, THF, or PyBOP, DIPEA (*N*,*N*-Diisopropylethylamine), DMF, r.t.

**Scheme 2:**
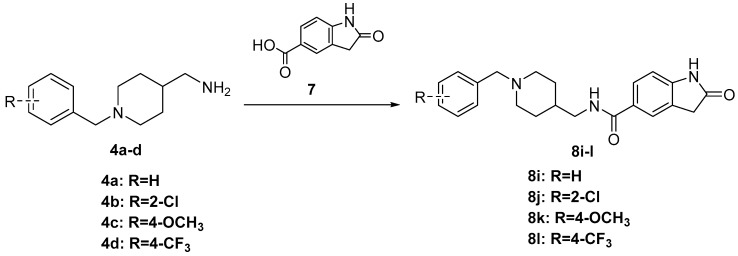
Synthesis of compounds **8i**–**l**. Reagents and conditions: 2-Oxoindoline-5-carboxylic acid 7, PyBOP, DIPEA, DMF, r.t. 24 h.

**Scheme 3:**
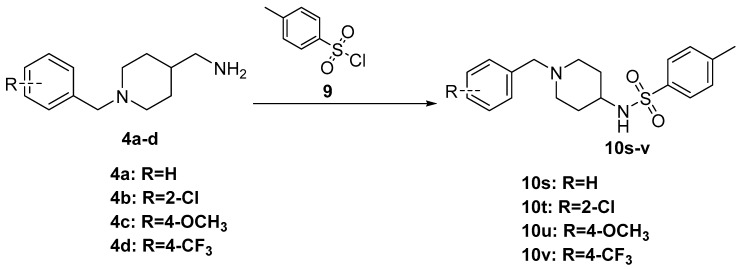
Synthesis of compounds **10s**–**v**. Reagents and conditions: TsCl **9**, Et_3_N, DCM, 0 °C 4 h.

The synthesis of the designed compounds **6a**–**h** and **8i**–**l** started from 4-piperidinecarboxamide **1**. At the first step, compound **1** was reacted with appropriate substituted Benzylchloride derivatives (**2a**–**d**) under potassium carbonate (K_2_CO_3_) and potassium iodide (KI) conditions, giving relevant N-Benzylpiperidin-4-carboxamide derivatives (**3a**–**d**) with medium yields (67%–81%). In addition, the obtained compounds **3a**–**d** were transformed into relevant substituted (1-Benzylpiperidin-4-yl) methanamine derivatives (**4a**–**d**) in the reduction reaction, which was carried out in dry tetrahydrofuran (THF) using lithium aluminum hydride (LiAlH_4_) under nitrogen atmosphere. The obtained compounds **4a**–**d** were used in further synthesis without purification. The yields of this step were between 74% and 82%. Finally, the commercially available nitrogen-containing heterocyclic aromatic carboxylic acids (**5a**–**d**) or 2-Oxoindoline-5-carboxylic acid **7** were activated with Carbonyldiimidazole (CDI) or Benzotriazol-1-yl-oxytripyrrolidinophosphonium hexafluorophosphate (PyBOP) and reacted with the appropriate amine (**4a**–**d**) in dry THF or *N*,*N*-dimethylformamide (DMF) to give the target compounds **6a**–**h** and **8i**–**l** with moderate to good yield (35%–80%) (Scheme 1 and Scheme 2). Compounds **10s**–**v** were prepared (73%–88% yield) by reactions of compounds **4a**–**d** with *p*-Toluenesulfonyl chloride (TsCl, **9**) under the presence of Triethylamine (Et_3_N) in Dichloromethane (DCM) (Scheme 3).

The structures of the new compounds were confirmed by spectral data (^1^H-NMR, ^13^C-NMR, and HRMS, see Supplementary Materials).

**Scheme 4:**
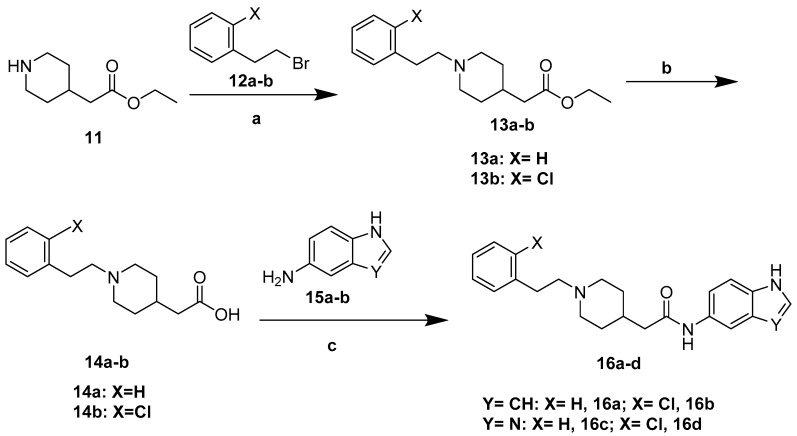
Synthesis of compounds **16a**–**d**. Reagents and conditions: (**a**) (2-Bromoethyl) benzene derivatives **12a**–**b**, K_2_CO_3_, catalytic amount of KI, acetone, reflux 4 h; (**b**) 4 mol/L KOH, C_2_H_5_OH: H_2_O = 5:1, r.t. 7 h; (**c**) compounds **15a**–**b**, PyBOP, DIPEA, DMF, r.t. 24 h.

The synthetic route of compounds **16a**–**d** has been depicted in Scheme 4. Ethyl 2-piperidin-4-ylacetate **11** was reacted with appropriate substituted (2-Bromoethyl) benzene derivatives (**12a**–**b**) under potassium carbonate (K_2_CO_3_) and potassium iodide (KI) conditions. Then, the obtained compounds **13a**–**b** were used in further synthesis without purification. 4 mol/L potassium hydroxide (KOH) was added to compounds **13a**–**b** in C_2_H_5_OH: H_2_O = 5:1. The reaction mixture was stirred at room temperature for 7 h to give compounds **14a**–**d.** Finally, compounds **14a**–**d** were activated with PyBOP and reacted with compounds **15a**–**b** in DMF to give the target compounds **16a**–**d** with moderate to good yield (30%–80%) (Scheme 4).

### 2.2. AChE and BChE Inhibitory Activity of the Target Molecules

Compounds **6a**–**h**, **8i**–**l**, **11a**–**f**, **10s**–**v**, and **16a**–**d** were evaluated for their anti-ChEs activity. Tacrine and Donepezil were used as reference drugs. According to the method described by Ellman [32], the data were expressed by IC_50_ values. In vitro experiments showed that some of these compounds could effectively inhibit ChEs in the micromolar range (Table 1).

**Figure 2:**
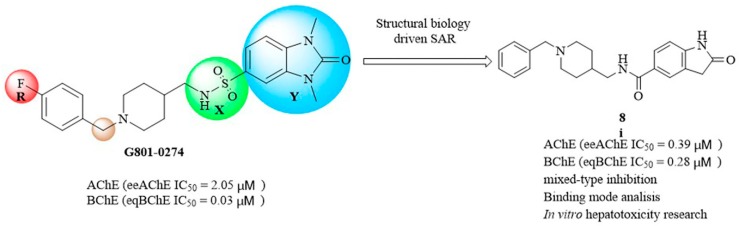
Design of compound **8i** for dual-target inhibitor of AChE and BChE.

First, we synthesized compounds **6a**, **6c**, **6d**, **8i** by maintaining the R as H, X as amide and exploring Y with 1*H*-Indole, 1*H*-Indazole, 1*H*-Benzo[*d*] imidazole or 2-Oxoindoline, together with the known analog **11f**.We found that when Y is substituted by a series of different structures, its activity on AChE are 2-Oxoindoline (**8i**) > 1*H*-Benzo[*d*] [1,2,3] triazole (**11f**) > 1*H*-Benzo[*d*]imidazole (**6d**) > 1*H*-Indazole (**6c**) > 1*H*-Indole (**6a**); its activity on BChE are 2-Oxoindoline (**8i**) > 1*H*-Benzo[*d*]imidazole (**6d**) > 1*H*-Indole (**6a**) > 1*H*-Benzo[*d*] [1,2,3] triazole (**11f**). Among them, compound **8i** (Figure 2) showed the strongest inhibitory effect on both AChE (eeAChE IC_50_ = 0.39 μM) and BChE (eqBChE IC_50_ = 0.28 μM), these results indicated that compound **8i** was a potent dual inhibitor against AChE and BChE. It was speculated that 2-Oxoindoline is the key structure for inhibiting two ChEs and is not selective for both ChEs. According to the molecular docking results, for eeAChE, 2-Oxoindoline of compound **8i** bound with Trp286 via π-π stacking interaction; for huAChE, 2-Oxoindoline of compound **8i** bound with Trp286 and Tyr341 via π-π stacking interaction, and for huBChE, 2-Oxoindoline of compound **8i** bound with Phe329 via π-π stacking interaction. These interactions increase the inhibitory activity by enhancing the binding affinity.

Then, we evaluated the effect of R on ChEs activity. We modified R with different substituent (2-Cl, 4-OCH_3_, 4-CF_3_), compared with compound **6d**, when R is 4-CF_3_ (**6e**), 4-OCH_3_ (**11d**), 2-Cl (**11e**), the ChEs inhibitory activity decreased. In particular, compound **11d** showed little inhibitory activity on BChE at 100 μM. In general, with the same Y, when R is H, the compounds have the highest inhibitory activity against ChEs; when Y is substituted by 2-Cl or 4-CF_3_, the inhibitory activity of the compounds to ChEs were weakened; and when Y is substituted by 4-OCH_3_, the compounds have the worst inhibitory activity against AChEs. We speculate that enhancing the electron-withdrawing effect or the donor effect on the aromatic ring is not conducive to improving the performance of the analog, and the appropriate space may facilitate the analog to enter the CAS pocket of ChEs.

Next, we investigated the effect of the *p*-Toluenesulfonamide moiety on the inhibitory activity of ChEs. Not only the amide group was replaced by Sulfonamide moiety based on the principles of bioisosterism, but also the fused nitrogen-containing bicyclic system (Indole, Indazole, Oxoindoline, Benzimidazole) in the previous compounds was replaced by Tosyl moiety. We synthesized compounds **10s**–**v**. Except for compound **10u** (eeAChE 11.61% [100 μM], eqBChE 18.31% [100 μM]) has low inhibitory activity against ChEs, the compounds **10s** (eeAChE IC_50_ = 4.24 μM, eqBChE IC_50_ = 4.10 μM)), **10t** (eeAChE IC_50_ = 5.50 μM, eqBChE IC_50_ = 2.01 μM), **10v** (eeAChE IC_50_ = 7.20 μM, eqBChE IC_50_ = 7.14 μM) all can maintain ChEs inhibitory activity at micromolar levels, indicating that the *p*-Toluenesulfonamide moiety is responsible for maintaining the inhibitory activity of ChEs. On one hand, we speculate that methyl occupies the pocket of the active site and interacts with amino acid residues to increase inhibitory activity. On the other hand, Sulfonamide moiety is very important for maintaining ChEs inhibitory activity, which may be related to the bond angle between Sulfonamide and molecules [33].

In addition, we tested the number of carbon atoms between benzene and piperidine. When the number of carbon atoms becomes two (compounds **16a**–**d**), the compounds had low inhibitory activity against the two kinds of ChEs, in particular, compound **16c** has no inhibitory activity against AChE at 100 μM. We speculate that the decrease in the activity of such compounds may be that the molecular volume is too large to enter the active pocket of ChEs, indicating that the residue of N-Benzylpiperidine in the structure is an essential group for inhibiting both ChEs.

Compounds designed with a Piperazine ring instead of a piperidine ring may also have the same or higher inhibitory activity on ChEs. Luca P, Tomás Daniel, Asha H, et al. [34] based on the structure of Donepezil, mainly the conjugation of Benzylpiperidine/ Benzylpiperazine moiety with a biologically active heterocyclic derivative (Benzimidazole or Benzofuran), which gave the compound other relevant properties. It shows good activity (${IC}_{50}^{1/4}$4.0–30.0 μM) for AChE inhibition, and has inhibition of Aβ peptide aggregation, antioxidant activity, and metal chelation.

### 2.3. Kinetic Studies of AChE and BChE Inhibition

**Figure 3:**
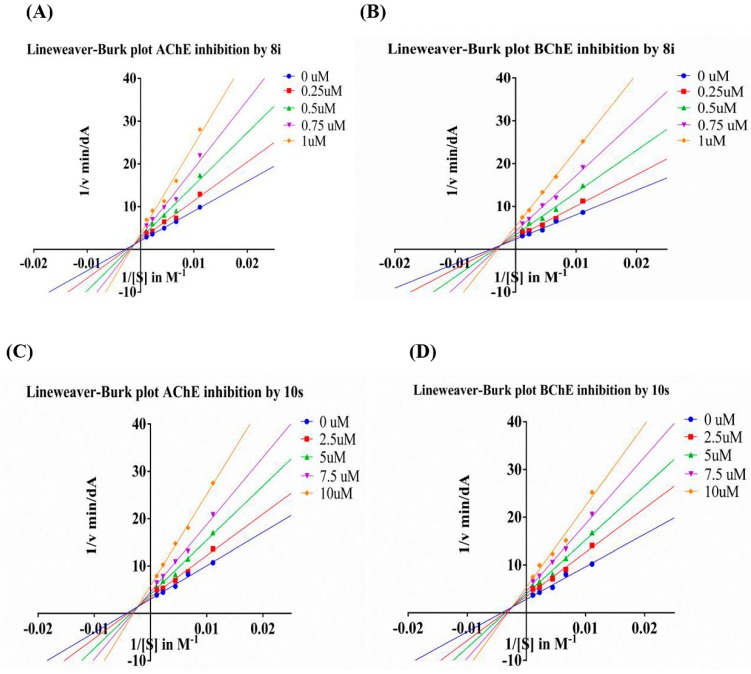
(**A**) Lineweaver–Burk plot for the inhibition of eeAChE (**A**) and eqBChE (**B**) by compound **8i** at different concentrations of substrate. (**B**) Lineweaver–Burk plot for the inhibition of eeAChE (**C**) and eqBChE (**D**) by compound **10s** at different concentrations of substrate.

To determine the kinetic types of AChE and BChE inhibition, compounds **8i** and **10s** were selected for kinetic studies. In each case, the kinetic types of enzyme inhibition were obtained by the modified Ellman’s method and the Lineweaver–Burk secondary plots [35]. The Lineweaver–Burk plots showed both increasing slope (decreased Vmax) and increasing intercept (higher Km) for higher inhibitor concentrations, indicating a mixed-type inhibition, including competitive inhibition and non-competitive inhibition, which possibly was because compound **8i** could bind to both CAS and PAS (Figure 3A,B). According to the result of molecular docking study. The same inhibition type between compound **10s** and ChEs was found in graphical analysis (Figure 3C,D).

### 2.4. Docking Studies

**Figure 4:**
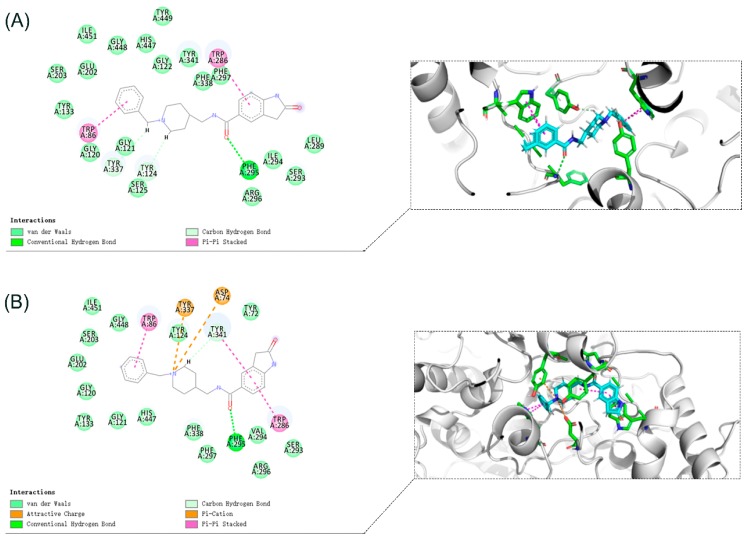
(**A**) Binding mode prediction of compound **8i** with eeAChE (PDB ID: 1C2B) (**B**) Binding mode prediction of compound **8i** with huAChE (PDB ID: 4EY7).

**Figure 5:**
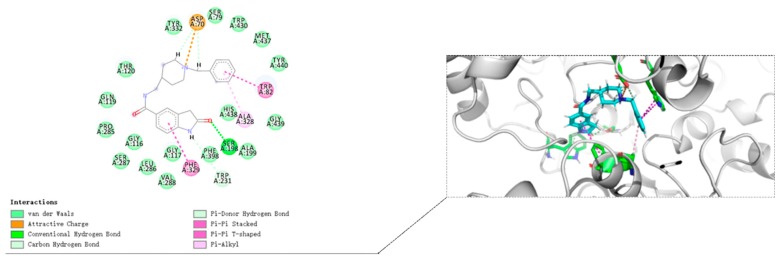
Binding mode prediction of compound **8i** with huBChE (PDB ID: 4TPK).

To further study the binding mode of compound **8i** and ChEs, molecular docking was performed using Discovery Studio software 2016. The predicted binding mode of compound **8i** is shown in Figure 4 and Figure 5. Compound **8i** could interact with CAS and PAS of AChE simultaneously. For AChE (from *Electrophorus electricus* (electric *ee*AChE, Sigma-Aldrich) (Figure 4A), the *N*-Benzylpiperidine moiety interacted with Trp86 in CAS via aromatic π-π interaction. The Amide group formed hydrogen bond with Phe295. Moreover, 2-Oxoindoline of compound **8i** bound with Trp286 via π-π stacking interaction; for human AChE-huAChE (Sigma-Aldrich) (Figure 4B), the N-Benzylpiperidine moiety of compound **8i** was bound to CAS, displaying a classic aromatic π-π interaction with Trp86. Moreover, 2-Oxoindoline of compound **8i** bound with Trp286 and Tyr341 via π-π stacking interaction. In addition, the Amide group formed hydrogen bond with Phe295. By comparison, it was found that the compounds have similar binding patterns to eeAChE and huAChE. All these facts provide an explanation for the higher inhibitory effects of compound **8i** towards AChE.

Molecular docking of compound **8i** at the active site of human BuChE-huBChE (Sigma-Aldrich, Munich, Germany) has been shown in Figure 5. The *N*-Benzylpiperidine moiety of compound **8i** interacts with Asp70 via electrostatic interaction. Moreover, the *N*-Benzylpiperidine moiety interacts with Trp82 in CAS by T-shaped π-π interaction, and the *N*-Benzylpiperidine moiety forms π-alkyl interaction with Ala328. 2-Oxoindoline moiety of compound **8i** bound with Phe329 via π-π stacking interaction. These interactions increase the inhibitory activity by enhancing the binding affinity.

**Figure 6:**
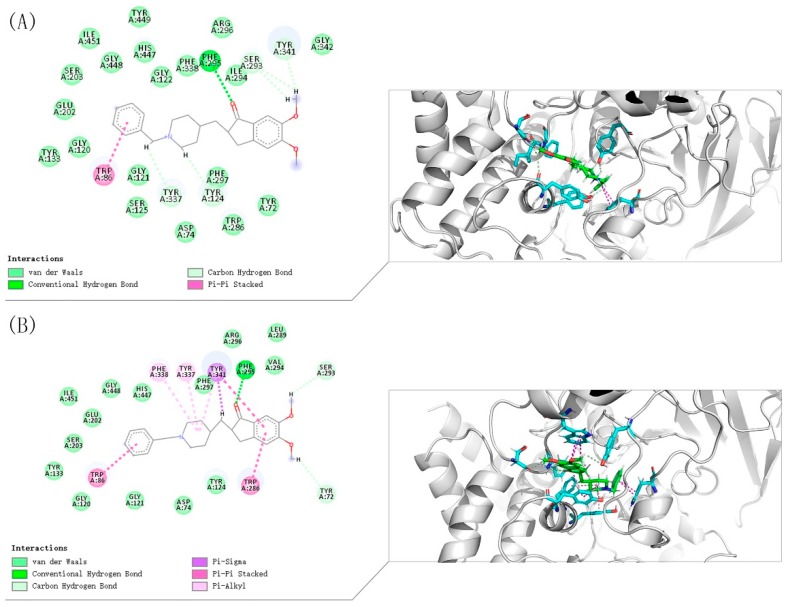
(**A**) Binding mode prediction of Donepezil with eeAChE (PDB ID: 1C2B); (**B**) Binding mode prediction of Donepezil with huAChE (PDB ID: 4EY7).

From the binding mode prediction of Donepezil with huAChE (Figure 6), we found that the binding pattern of compound **8i** is similar to Donepezil in some respects: (i) The *N*-Benzylpiperidine moiety was bound to CAS, displaying a classic aromatic π-π interaction with Trp86; (ii) The Oxygen atom formed hydrogen bond with Phe295; (iii) Aromatic heterocycle moiety bound with Trp286 and Tyr341 via π-π stacking interaction. In addition, the Indone moiety of Donepezil interacts with Trp286 at the center of the PAS; the piperidine moiety of Donepezil interacts with Tyr337, Phe338, Tyr341.

**Figure 7:**
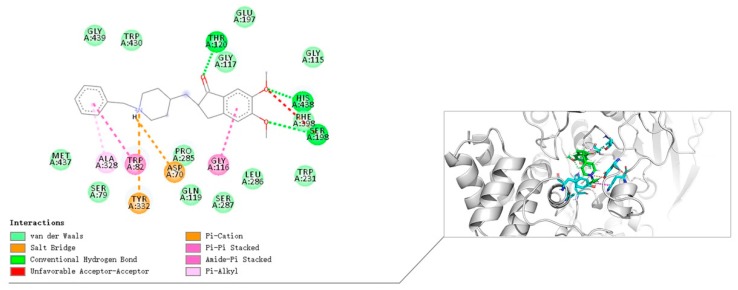
Binding mode prediction of Donepezil with huBChE (PDB ID: 4TPK).

From the binding mode prediction of Donepezil with huBChE (Figure 7), we also found that the binding pattern of compound **8i** is similar to Donepezil in some respects: (i) The *N*-Benzylpiperidine moiety interacts with Asp70 via electrostatic interaction. In addition, the *N*-Benzylpiperidine moiety of Donepezil interacts with Try332 via electrostatic interaction; (ii) the *N*-Benzylpiperidine moiety interacts with Trp82 in CAS by T-shaped π-π interaction, and the *N*-Benzylpiperidine moiety form π-alkyl interaction with Ala328. The difference is that 2-Oxoindoline of compound **8i** bound with Phe329 via π-π stacking interaction while the 2-Oxoindoline moiety of Donepezil interacts with Gly116 in the center of the PAS.

Compared with Donepezil, the inhibitory activity of compound **8i** on BChE is higher than Donepezil, and has similar inhibitory activity on two ChEs, so that it can exert an anti-ChEs effect in a balanced manner. Studies on compound **8i** molecular docking have shown that the Benzylpiperidine moiety of the compound acts on the CAS of the enzyme, while the 2-Oxoindoline moiety binds to the PAS of the enzyme, which is basically consistent with the design idea.

### 2.5. Cytotoxicity Studies

**Figure 8:**
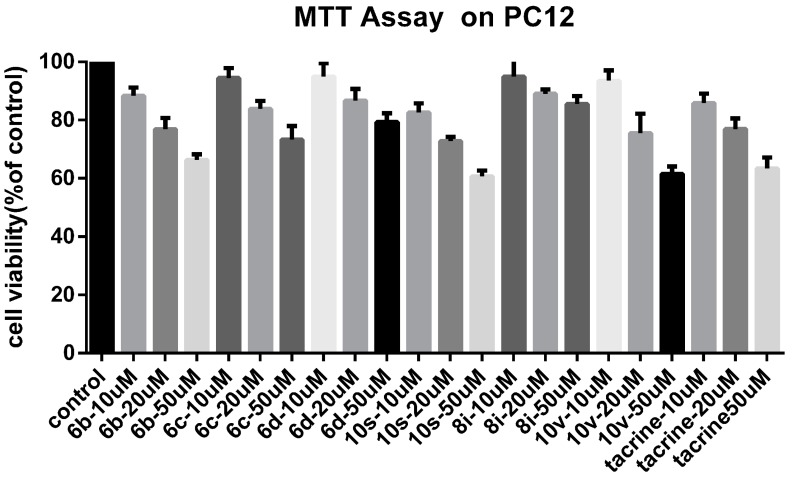
In vitro cell toxicity of compounds **6b**, **6c**, **6d**, **10s**, **8i**, and **10v** on PC-12 cell line. Data were expressed as mean ± SD (*n* = 3).

We focused on the cytotoxicity of the synthetic compounds. The reason for using PC12 is that our compounds act on the central nervous system, so we need to find out whether the compound has a toxic effect on normal nerve cells. Compounds **6b**, **6c**, **6d**, **10s**, **8i**, and **10v** were selected as representative compounds to assess their potential cytotoxic effects. Compounds **6c**, **6d**, **8i** are less toxic than Tacrine; the toxicity of compounds **6b**, **10v** are similar to that of Tacrine; compound **10s** is slightly more toxic than Tacrine. Among them, compound **8i** has the lowest toxicity (Figure 8).

## 3. Materials and Methods

### 3.1. Chemistry

All reagents were obtained from commercial suppliers and were used without any further purification unless otherwise stated. Flash column chromatography was performed with silica gel (200-300 mesh) purchased from Qingdao Haiyang Chemical Co. Ltd. Thin layer chromatography was performed using silica gel 60 F254 precoated plates (purchased from Qingdao Haiyang Inc., Qingdao, China). Visualization was achieved using Ultraviolet (UV) light (254 nm and 365 nm, Shanghai Yarong Biochemical Instrument Factory, Shanghai, China). Melting points were determined with a Mel-TEMP II melting point apparatus (Beijing Keyi Company, Beijing, China) and was uncorrected. ^1^H NMR and ^13^C NMR spectra were recorded with Bruker AV-600, AV-500 or AV-400 MHz instruments (Bruker, Ettlingen, Germany) using DMSO-*d*_6_, CD_3_OD, or CDCl_3_ as solvent. Chemical shifts were reported as δ values (ppm) from internal reference tetramethylsilane (TMS). All coupling constants were reported in hertz (Hz), All chemical shifts are reported in parts per million (ppm), relative to the internal standard. In addition, proton multiplicities were labeled as br (broad), s (singlet), d (doublet), dd (doublet of doublets), t (triplet), q (quartet), and m (multiplet). HR-MS were performed on a Waters Vion IMS Q-tof (Waters, MA, USA).

### 3.2. General Procedure for the Synthesis of Compounds ***3a**–**d***

4-Piperidinecarboxamide (**1**) (3.00 g, 23.4 mmol) and substituted Benzylchloride derivatives (**2a**–**d**) (28.1 mmol) were dissolved in 20 mL acetone. Then, anhydrous K_2_CO_3_ (6.47 g, 46.8 mmol) and catalytic amount KI were added. The reaction mixture was refluxed for 4 h. After completion of the reaction, acetone was concentrated, and the residue was dissolved in water (60 mL) and extracted with ethyl acetate (60 × 3 mL). The combined organic layers were dried over Na_2_SO_4_, filtered, and the solvent was removed under reduced pressure. After concentration, the crude product was purified by silica gel column chromatograph (DCM: methanol = 60:1–5:1) to give target compounds **3a**–**d**.

### 3.3. General Procedure for the Synthesis of Compounds ***4a**–**d***

Compounds **3a**–**d** (3 g) were dissolved in anhydrous THF (17 mL) and then LiAlH_4_ (5 equiv.) was added to the above cooled solution at 0–5 °C in small portions under stirring. The reaction mixture was further stirred at room temperature for 30 min and finally refluxed for 4 h. After cooling, water and 10% NaOH solution was added at 0–5 °C. Then, the obtained white precipitate was filtered off and washed with THF. The filtrate was extracted with ethyl acetate. The combined organic layers were dried over anhydrous Na_2_SO_4_, filtered, and concentrated in vacuum. The obtained compounds **4a**–**d** were used in further synthesis without purification.

### 3.4. General Procedure for the Synthesis of Compounds ***6a**–**h***

CDI (1 equiv.) was added to a solution of the acids (**5a**–**d**) ((300 mg**,** 1 equiv.) in dry THF under nitrogen atmosphere. After 30 min, the solution of substituted 4-Amine-1-benzylpiperidines (**4a**–**d**) (1.2 equiv.) in THF were added, and the reaction mixture was stirred at room temperature for 24 h. After the reaction was completed, the solvent was removed under reduced pressure, and then the reaction mixture was quenched with saturated NaCl solution (25 mL). The aqueous phase was extracted with DCM (25 × 3 mL). The DCM layer was combined and washed with brine solution (25 × 3 mL). The organic layer was dried over anhydrous Na_2_SO_4_ and the solvent was removed under reduced pressure. After concentration, the crude product was purified by silica gel column chromatograph (DCM: methanol = 60:1–5:1) to give target compounds **6a**–**h**.

*N-((1-Benzylpiperidin-4-yl)methyl)-1H-indole-5-carboxamide* (**6a**). 1*H*-Indole-5-carboxylic acid (300 mg, 1.86 mmol), CDI (302 mg, 1.86 mmol), (1-Benzylpiperidin-4-yl)methanamine (457 mg, 2.24 mmol), THF (15 mL) White solid, m.p.: 89–90 °C, yield: 80%, ^1^H NMR (500 MHz, DMSO-*d*_6_) δ 11.50 (s, 1H), 8.47 (s, 1H), 8.17 (s, 1H), 7.66 (d, *J* = 8.4 Hz, 1H), 7.45 (d, *J* = 8.6 Hz, 1H), 7.41 (d, *J* = 7.1 Hz, 2H), 7.36–7.29 (m, 3H), 6.51 (s, 1H), 3.78 (s, 2H), 3.19 (d, *J* = 5.5 Hz, 2H), 2.99 (d, *J* = 10.8 Hz, 2H), 2.31 (d, *J* = 11.0 Hz, 2H), 1.72 (d, *J* = 12.0 Hz, 3H), 1.38 (d, *J* = 11.4 Hz, 2H). ^13^C NMR (126 MHz, DMSO-*d*_6_) δ 168.15, 137.84, 135.07, 130.40, 128.81, 128.39, 127.44, 127.05, 125.94, 120.97, 120.37, 111.37, 102.49, 61.15, 52.37, 44.70, 35.20, 28.63. HRMS (ESI): calcd. For C_22_H_25_N_3_O [M + H]^+^ 348.2070, found 348.2070.

*N-((1-(2-Chlorobenzyl)piperidin-4-yl)methyl)-1H-Indole-5-carboxamide* (**6b**). 1*H*-Indole-5-carboxylic acid (300 mg, 1.86 mmol), CDI (302 mg, 1.86 mmol), (1-(2-Chlorobenzyl) piperidin-4-yl)methanamine (532 mg, 2.24 mmol), THF (15 mL) White solid, m.p.: 88–89 °C, yield: 66%, ^1^H NMR (400 MHz, CD_3_OD) δ 8.08 (d, *J* = 1.1 Hz, 1H), 7.58 (dd, *J* = 8.6, 1.8 Hz, 1H), 7.49 (dd, *J* = 7.1, 2.2 Hz, 1H), 7.41–7.35 (m, 2H), 7.30–7.24 (m, 3H), 6.51 (d, *J* = 3.2 Hz, 1H), 3.78 (s, 2H), 3.05 (d, *J* = 11.7 Hz, 2H), 2.31 (t, *J* = 11.9 Hz, 2H), 1.77 (t, *J* = 14.0 Hz, 3H), 1.45–1.33 (m, 2H). ^13^C NMR (101 MHz, CD_3_OD) δ 170.60, 138.15, 134.64, 131.71, 129.37, 129.10, 127.70, 126.78, 125.91, 125.17, 120.18, 119.95, 110.67, 102.17, 58.57, 53.06, 44.79, 35.56, 28.93. HRMS (ESI): calcd. For C_22_H_24_ClN_3_O [M + H]^+^ 382.1681, found 382.1703.

*N-((1-Benzylpiperidin-4-yl) methyl)-1H-indazole-5-carboxamide* (**6c**). 1*H*-Indazole-5-carboxylic acid (300 mg, 1.86 mmol), CDI (302 mg, 1.86 mmol), (1-Benzylpiperidin-4-yl)methanamine (457 mg, 2.24 mmol), THF (15 mL) White solid, m.p.: 111–112 °C, yield: 56%, ^1^H NMR (500 MHz, DMSO-*d*_6_) δ 13.30 (s, 1H), 8.48 (s, 1H), 8.35 (s, 1H), 8.20 (s, 1H), 7.87 (d, *J* = 8.8 Hz, 1H), 7.57 (d, *J* = 8.7 Hz, 1H), 7.33–7.27 (m, 3H), 7.24 (d, *J* = 6.8 Hz, 1H), 3.45 (s, 2H), 3.18 (d, *J* = 6.0 Hz, 2H), 2.81 (d, *J* = 11.1 Hz, 2H), 1.92 (t, *J* = 11.1 Hz, 2H), 1.67 (d, *J* = 12.4 Hz, 2H), 1.59 (s, 1H), 1.22 (d, *J* = 9.4 Hz, 2H). ^13^C NMR (126 MHz, DMSO-*d*_6_) δ 167.07, 141.33, 138.83, 135.11, 129.24, 128.56, 127.67, 127.62, 127.30, 125.79, 122.79, 120.90, 110.16, 62.78, 53.38, 45.31, 36.17, 30.23. HRMS (ESI): calcd. For C_21_H_24_N_4_O [M + H]^+^ 349.2023, found 349.2019.

*N-((1-Benzylpiperidin-4-yl)methyl)-1H-benzo[d]imidazole-5-carboxamide* (**6d**). 1*H*-Benzo[*d*]imidazole-5-carboxylic acid (300 mg, 1.85 mmol), CDI (300 mg, 1.85 mmol), (1-Benzylpiperidin-4-yl)methanamine (453 mg, 2.22 mmol), THF (15 mL) Yellow oil, yield: 72%, ^1^H NMR (500 MHz, DMSO-*d*_6_) δ 8.53 (s, 1H), 8.27 (d, *J* = 68.8 Hz, 1H), 7.78 (d, *J* = 8.0 Hz, 1H), 7.70–7.61 (m, 1H), 7.28 (d, *J* = 6.9 Hz, 3H), 7.21 (d, *J* = 5.6 Hz, 1H), 7.04 (s, 1H), 3.39 (s, 2H), 3.19 (s, 2H), 2.76 (s, 2H), 1.85 (d, *J* = 11.8 Hz, 2H), 1.65 (d, *J* = 11.8 Hz, 3H), 1.20 (d, *J* = 10.9 Hz, 2H). ^13^C NMR (126 MHz, DMSO-*d*_6_) δ 167.40, 144.24, 139.08, 135.64, 129.16, 128.51, 127.18, 121.88, 115.66, 114.85, 62.93, 53.43, 53.35, 45.39, 36.25, 30.32. HRMS (ESI): calcd. For C_21_H_24_N_4_O [M + H]^+^ 349.2023, found 349.2023.

*N-((1-(4-(Trifluoromethyl)benzyl)piperidin-4-yl)methyl)-1H-benzo[d]imidazole-5-carboxamide* (**6e**). 1*H*-Benzo[*d*]imidazole-5-carboxylic acid (300 mg, 1.85 mmol), CDI (300 mg, 1.85 mmol), (1-(4-(Trifluoromethyl)benzyl)piperidin-4-yl)methanamine (604 mg, 2.22 mmol), THF (15 mL) White solid, m.p.: 92–93 °C, yield: 77%, ^1^H NMR (400 MHz, CD_3_OD) δ 8.29–8.27 (m, 1H), 8.12 (d, *J* = 1.0 Hz, 1H), 7.87–7.77 (m, 2H), 7.62 (d, *J* = 7.8 Hz, 1H), 7.56 (t, *J* = 8.9 Hz, 2H), 7.39 (p, *J* = 5.5 Hz, 2H), 3.66 (d, *J* = 6.7 Hz, 2H), 3.30 (s, 1H), 3.27 (s, 1H), 2.90 (d, *J* = 11.4 Hz, 2H), 2.15–2.06 (m, 2H), 1.79–1.63 (m, 3H), 1.44–1.24 (m, 3H). ^13^C NMR (126 MHz, CD_3_OD) δ 169.23, 141.32, 134.81, 131.92, 130.75, 130.34, 128.00, 127.37, 127.30, 125.41, 122.55, 120.67, 114.13, 113.58, 109.69, 58.94, 57.96, 53.34, 53.01, 44.93, 44.83, 35.64, 35.52, 29.26, 29.06. HRMS (ESI): calcd. For C_22_H_23_F_3_N_4_O [M + H]^+^ 417.1897, found 417.1895.

*N-((1-(4-Methoxybenzyl)piperidin-4-yl)methyl)-1H-benzo[d][1,2,3]triazole-5-carboxamide* (**6f**). 1*H*-Benzo[*d*][1,2,3]triazole-5-carboxylic acid (300 mg, 1.84 mmol), CDI (298 mg, 1.84 mmol), (1-(4-Methoxybenzyl)piperidin-4-yl)methanamine (517 mg, 2.21 mmol), THF (15 mL) Yellow oil, yield: 63%, ^1^H NMR (500 MHz, DMSO-*d*_6_) δ 8.53 (s, 1H), 8.32 (s, 1H), 8.16 (s, 1H), 7.75 (d, *J* = 8.4 Hz, 1H), 7.61 (d, *J* = 8.3 Hz, 1H), 7.32 (d, *J* = 8.3 Hz, 2H), 6.91 (d, *J* = 8.3 Hz, 2H), 3.74 (s, 3H), 3.69 (s, 2H), 3.18 (d, *J* = 5.9 Hz, 2H), 2.97 (d, *J* = 11.1 Hz, 2H), 2.26 (s, 2H), 1.73 (d, *J* = 13.0 Hz, 2H), 1.68 (s, 1H), 1.34 (d, *J* = 11.2 Hz, 2H). ^13^C NMR (126 MHz, DMSO-*d*_6_) δ 167.31, 159.33, 144.22, 131.57, 128.92, 121.94, 114.16, 60.78, 55.52, 52.34, 44.84, 35.34, 28.86. HRMS (ESI): calcd. For C_21_H_25_N_5_O_2_ [M + H]^+^ 380.2081, found 380.2077.

*N-((1-(2-Chlorobenzyl)piperidin-4-yl)methyl)-1H-benzo[d][1,2,3]triazole-5-carboxamide* (**6g**). 1*H*-Benzo[*d*][1,2,3]triazole-5-carboxylic acid (300 mg, 1.84 mmol), CDI (298 mg, 1.84 mmol), (1-(2-Chlorobenzyl)piperidin-4-yl)methanamine (526 mg, 2.21 mmol), THF (15 mL) White solid, m.p.: 77–79 °C, yield: 57%, ^1^H NMR (500 MHz, DMSO-*d*_6_) δ 8.63 (s, 1H), 8.44 (s, 1H), 7.87 (d, *J* = 2.7 Hz, 2H), 7.47 (d, *J* = 7.1 Hz, 1H), 7.39 (d, *J* = 7.8 Hz, 1H), 7.30 (d, *J* = 6.9 Hz, 1H), 7.27–7.25 (m, 1H), 7.03 (s, 1H), 3.52 (s, 2H), 3.21 (t, *J* = 6.1 Hz, 2H), 2.82 (d, *J* = 11.3 Hz, 2H), 2.00 (t, *J* = 10.4 Hz, 2H), 1.68 (d, *J* = 12.0 Hz, 2H), 1.61 (s, 1H), 1.23 (d, *J* = 10.5 Hz, 2H). ^13^C NMR (126 MHz, DMSO-*d*_6_) δ 166.50, 140.30, 139.38, 138.70, 131.96, 129.27, 128.56, 127.32, 125.46, 115.61, 114.32, 62.73, 53.33, 45.41, 36.08, 30.16. HRMS (ESI): calcd. For C_20_H_22_ClN_5_O [M + H]^+^ 384.1586, found 384.1584.

*N-((1-(4-(Trifluoromethyl)benzyl)piperidin-4-yl)methyl)-1H-benzo[d][1,2,3]triazole-5-carboxamide* (**6h**). 1*H*-Benzo[*d*][1,2,3]triazole-5-carboxylic acid (300 mg, 1.84 mmol), CDI (298 mg, 1.84 mmol), (1-(4-(Trifluoromethyl)benzyl)piperidin-4-yl)methanamine (601 mg, 2.21 mmol), THF (15 mL) White solid, m.p.: 90–91 °C, yield: 73%, ^1^H NMR (400 MHz, CD_3_OD) δ 8.38 (td, *J* = 1.5, 1.0 Hz, 1H), 7.92–7.90 (m, 1H), 7.88 (t, *J* = 1.0 Hz, 1H), 7.81 (d, *J* = 7.8 Hz, 1H), 7.68 (d, *J* = 7.8 Hz, 1H), 7.61 (dt, *J* = 7.7, 4.1 Hz, 1H), 7.51–7.42 (m, 2H), 3.89 (d, *J* = 22.6 Hz, 2H), 3.33 (dd, *J* = 6.5, 3.7 Hz, 2H), 3.13–3.01 (m, 2H), 2.47–2.31 (m, 2H), 1.90–1.68 (m, 3H), 1.52–1.32 (m, 2H). ^13^C NMR (126 MHz, DMSO-*d*_6_) δ 166.50, 140.30, 139.38, 138.70, 131.96, 129.27, 128.56, 127.32, 125.46, 115.61, 114.32, 62.73, 53.33, 45.41, 36.08, 30.16. HRMS (ESI): calcd. For C_21_H_22_F_3_N_5_O [M + H]^+^ 418.1849, found 418.1850.

### 3.5. General Procedure for the Synthesis of ***8i**–**l***

Intermediates (**7**) (140 mg, 1.2 equiv.), PyBOP (1.2 equiv.) and DIPEA (1.5 equiv.) were added to DMF and stirred at room temperature for 20 min. Then, intermediates (**4a**–**d**) (1.0 equiv.) was added and stirred at room temperature for 4 h. After completion of the reaction, the reaction mixture was quenched with saturated NaCl solution (25 mL). The aqueous phase was extracted with DCM (25 × 3 mL). The DCM layer was combined and washed with brine solution (25 × 3 mL). The organic layer was dried over anhydrous Na_2_SO_4_ and the solvent was removed under reduced pressure. After concentration, the crude product was purified by silica gel column chromatograph using a methanol in DCM gradient (DCM: methanol= 60:1–5:1) yielded compounds **8i**–**l**.

*N-((1-Benzylpiperidin-4-yl)methyl)-2-Oxoindoline-5-carboxamide* (**8i**). 2-Oxoindoline-5-carboxylic acid (140 mg, 0.79 mmol), (1-Benzylpiperidin-4-yl)methanamine (135 mg, 0.66 mmol), PyBOP (412 mg, 0.79 mmol), DIPEA(128 mg, 0.99 mmol), DMF (6 mL). White solid, m.p.:155–156 °C, yield: 56%, ^1^H NMR (400 MHz, CD_3_OD) δ 7.73–7.68 (m, 2H), 7.53–7.42 (m, 5H), 6.91 (d, *J* = 8.1 Hz, 1H), 4.26 (s, 2H), 3.44 (d, *J* = 12.5 Hz, 2H), 3.31 (s, 1H), 2.98 (t, *J* = 12.9 Hz, 2H), 1.97 (d, *J* = 13.6 Hz, 3H), 1.54 (q, *J* = 13.1, 12.1 Hz, 2H). ^13^C NMR (101 MHz, CD_3_OD) δ 178.52, 168.90, 146.69, 130.99, 129.80, 129.40, 128.99, 127.92, 127.60, 125.95, 123.42, 123.40, 108.97, 60.21, 51.86, 43.82, 42.11, 34.01, 26.82. HRMS (ESI): calcd. For C_22_H_25_N_3_O_2_ [M + H]^+^ 364.2020, found 364.2032.

*N-((1-(2-Chlorobenzyl)piperidin-4-yl)methyl)-2-Oxoindoline-5-carboxamide* (**8j**). 2-Oxoindoline-5-carboxylic acid (140 mg, 0.79 mmol), (1-(2-Chlorobenzyl)piperidin-4-yl)methanamine (157 mg, 0.66 mmol), PyBOP (412 mg, 0.79 mmol), DIPEA(128 mg, 0.99 mmol), DMF (6 mL). White solid, m.p.: 151–152 °C, yield: 35%, ^1^H NMR (600 MHz, CD_3_OD) δ 7.74–7.69 (m, 2H), 7.50 (d, *J* = 7.2 Hz, 1H), 7.39 (d, *J* = 7.6 Hz, 1H), 7.28 (dt, *J* = 20.1, 7.1 Hz, 2H), 6.93 (d, *J* = 8.1 Hz, 1H), 3.71 (s, 2H), 3.27 (d, *J* = 6.8 Hz, 2H), 3.00 (s, 2H), 2.21 (s, 2H), 1.76 (d, *J* = 12.7 Hz, 2H), 1.69 (s, 1H), 1.37 (q, *J* = 11.5 Hz, 2H). ^13^C NMR (101 MHz, CD_3_OD) δ 168.83, 146.63, 135.02, 132.40, 130.20, 129.68, 128.07, 127.58, 127.18, 125.93, 123.41, 108.96, 57.94, 52.76, 44.36, 34.83, 28.01. HRMS (ESI): calcd. For C_22_H_24_ClN_3_O_2_ [M + H]^+^ 398.1630, found 398.1652.

*N-((1-(4-Methoxybenzyl)piperidin-4-yl)methyl)-2-Oxoindoline-5-carboxamide* (**8k**). 2-Oxoindoline-5-carboxylic acid (140 mg, 0.79 mmol), (1-(4-Methoxybenzyl)piperidin-4-yl)methanamine (154 mg, 0.66 mmol), PyBOP (412 mg, 0.79 mmol), DIPEA(128 mg, 0.99 mmol), DMF (6 mL). White solid, m.p.: 158–159 °C, yield: 43%, ^1^H NMR (400 MHz, CD_3_OD) δ 7.74–7.68 (m, 2H), 7.40 (d, *J* = 8.7 Hz, 2H), 6.98 (d, *J* = 8.7 Hz, 2H), 6.91 (d, *J* = 8.0 Hz, 1H), 4.19 (s, 2H), 3.79 (s, 3H), 3.43 (d, *J* = 12.0 Hz, 2H), 3.31 (s, 1H), 2.95 (t, *J* = 11.7 Hz, 2H), 1.97 (d, *J* = 13.9 Hz, 3H), 1.60–1.45 (m, 2H), 1.26 (s, 1H). ^13^C NMR (101 MHz, CD_3_OD) δ 178.54, 168.87, 161.10, 146.66, 132.51, 127.95, 127.62, 125.93, 123.46, 121.09, 114.21, 108.98, 54.55, 51.51, 43.78, 34.03, 27.22, 26.85. HRMS (ESI): calcd. For C_23_H_27_N_3_O_3_ [M + H]^+^ 394.2125, found 394.2131.

*2-Oxo-N-((1-(4-(trifluoromethyl)benzyl)piperidin-4-yl)methyl)indoline-5-carboxamide* (**8l**). 2-Oxoindoline-5-carboxylic acid (140 mg, 0.79 mmol), (1-(4-(Trifluoromethyl)benzyl)piperidin-4-yl)methanamine (179 mg, 0.65 mmol), PyBOP (412 mg, 0.79 mmol), DIPEA(128 mg, 0.99 mmol), DMF (6 mL). White solid, m.p.: 113–115 °C, yield: 40%, ^1^H NMR (400 MHz, CD_3_OD) δ 7.79 (d, *J* = 7.8 Hz, 1H), 7.73–7.69 (m, 2H), 7.62 (d, *J* = 8.2 Hz, 1H), 7.56 (t, *J* = 7.8 Hz, 1H), 7.44–7.32 (m, 2H), 6.95–6.90 (m, 1H), 3.60 (d, *J* = 17.7 Hz, 2H), 3.24 (dd, *J* = 9.1, 6.8 Hz, 2H), 2.91–2.77 (m, 2H), 2.09–1.93 (m, 2H), 1.78–1.61 (m, 3H), 1.40–1.21 (m, 3H). ^13^C NMR (126 MHz, CD_3_OD)) δ 168.70, 146.44, 131.73, 130.35, 130.30, 128.23, 127.47, 127.30, 126.79, 125.81, 125.26, 123.34, 108.90, 59.79, 59.79, 58.24, 53.43, 53.14, 45.07, 36.04, 36.04, 36.00, 36.00, 29.82, 29.76. HRMS (ESI): calcd. For C_23_H_24_F_3_N_3_O_2_ [M + H]^+^ 432.1893, found 432.1886.

### 3.6. General Procedure for the Synthesis of ***10s**–**v***

To a solution of (1-Benzylpiperidin-4-yl) methanamine derivatives (**4a**–**d**) (150 mg, 1 equiv.) in DCM (5 mL) was added Et_3_N (1.8 equiv.). The mixture was cooled to 0 °C, TsCl (1.3 equiv.) in DCM (5 mL) was added dropwise, and the reaction mixture was stirred for an additional 1 h at 0 °C. After completion of the reaction, the reaction mixture was dissolved in 15 mL saturated sodium bicarbonate (NaHCO_3_) and extracted with DCM (25 × 3 mL). Organic phases were combined and washed with saturated NaCl solution (25 × 3 mL). The organic layer was dried over anhydrous Na_2_SO_4_ and the solvent was removed under reduced pressure. After concentration, the crude product was purified by silica gel column chromatograph using a methanol in dichloromethane gradient (dichloromethane: methanol = 60:1–5:1) yielded compounds **10s**–**v**.

*N-((1-Benzylpiperidin-4-yl)methyl)-4-methylbenzenesulfonamide* (**10s**). (1-Benzylpiperidin-4-yl) methanamine (150 mg, 0.73 mmol), TsCl (181 mg, 0.95 mmol), Et_3_N (133 mg, 1.31 mmol), DCM (9 mL) White solid, m.p.:78–79 °C, yield: 80%, ^1^H NMR (400 MHz, CD_3_OD) δ 7.68 (d, *J* = 8.3 Hz, 2H), 7.36–7.24 (m, 7H), 3.60 (s, 2H), 2.93 (d, *J* = 12.0 Hz, 2H), 2.67 (d, *J* = 6.8 Hz, 2H), 2.39 (s, 3H), 2.09 (t, *J* = 12.6 Hz, 2H), 1.68 (d, *J* = 13.8 Hz, 2H), 1.44 (s, 1H), 1.28–1.13 (m, 2H). ^13^C NMR (101 MHz, CD_3_OD) δ 143.22, 137.71, 136.09, 129.69, 129.36, 128.05, 127.39, 126.67, 62.59, 52.72, 35.66, 28.79, 20.11. HRMS (ESI): calcd. For C_20_H_26_N_2_O_2_S [M + H]^+^ 359.1788, found 359.1802.

*N-((1-(2-Chlorobenzyl)piperidin-4-yl)methyl)-4-methylbenzenesulfonamide* (**10t**). (1-(4-Chlorobenzyl)piperidin-4-yl)methanamine (150 mg,0.63 mmol), TsCl (156 mg, 0.82 mmol), Et_3_N (114 mg, 1.13 mmol), DCM (9 mL) White solid, m.p.: 111–112 °C, yield: 73%, ^1^H NMR (400 MHz, CD_3_OD) δ 7.69 (d, *J* = 8.3 Hz, 2H), 7.44 (dd, *J* = 7.4, 2.0 Hz, 1H), 7.37–7.31 (m, 3H), 7.23 (td, *J* = 7.1, 1.9 Hz, 2H), 3.62 (s, 2H), 2.89 (d, *J* = 11.8 Hz, 2H), 2.67 (d, *J* = 6.8 Hz, 2H), 2.39 (s, 3H), 2.06 (t, *J* = 11.7 Hz, 2H), 1.65 (d, *J* = 13.1 Hz, 2H), 1.37 (s, 1H), 1.20 (dd, *J* = 12.3, 3.6 Hz, 1H), 1.15 (s, 1H). ^13^C NMR (101 MHz, CD_3_OD) δ 143.42, 137.49, 135.59, 133.53, 131.79, 130.10, 129.48, 127.74, 127.35, 126.68, 126.68, 20.13. HRMS (ESI): calcd. For C_20_H_25_ClN_2_O_2_S [M + H]^+^ 393.1398, found 393.1397.

*N-((1-(4-Methoxybenzyl)piperidin-4-yl)methyl)-4-methylbenzenesulfonamide* (**10u**). (1-(4-Methoxybenzyl)piperidin-4-yl)methanamine (150 mg, 0.64 mmol), TsCl (158 mg, 0.83 mmol), Et_3_N (117 mg, 1.15 mmol), DCM (9 mL) White solid, m.p.: 76–77 °C, yield: 87%, ^1^H NMR (600 MHz, CD_3_OD) δ 7.61 (d, *J* = 8.3 Hz, 2H), 7.25 (dd, *J* = 27.4, 8.3 Hz, 4H), 6.85 (d, *J* = 8.6 Hz, 2H), 3.80 (s, 2H), 3.70 (s, 3H), 3.10 (d, *J* = 10.7 Hz, 2H), 2.63 (d, J = 6.7 Hz, 2H), 2.44 (s, 2H), 2.32 (s, 3H), 2.05 (s, 1H), 1.73 (s, 2H), 1.52 (s, 1H), 1.27–1.19 (m, 2H). ^13^C NMR (101 MHz, CD_3_OD) δ 160.41, 143.33, 137.60, 131.82, 129.42, 126.66, 113.87, 60.67, 54.47, 51.88, 34.72, 27.47, 20.10. HRMS (ESI): calcd. For C_21_H_28_N_2_O_3_S [M + H]^+^ 389.1893, found 389.1894.

*4-Methyl-N-((1-(4-(trifluoromethyl)benzyl)piperidin-4-yl)methyl)benzenesulfonamide* (**10v**). (1-(4-(Trifluoromethyl)benzyl)piperidin-4-yl)methanamine (150 mg, 0.55 mmol), TsCl (136 mg, 0.72 mmol), Et_3_N (100 mg, 0.99 mmol), DCM (9 mL) White solid, m.p.: 87–88 °C, yield: 88%, ^1^H NMR (400 MHz, CD_3_OD) δ 7.76 (d, *J* = 7.8 Hz, 1H), 7.71–7.66 (m, 2H), 7.61 (d, *J* = 7.9 Hz, 1H), 7.57–7.51 (m, 1H), 7.40–7.31 (m, 4H), 3.58 (d, *J* = 14.0 Hz, 2H), 2.79 (t, *J* = 12.2 Hz, 2H), 2.67 (t, *J* = 6.9 Hz, 2H), 2.40 (s, 3H), 1.97 (t, *J* = 11.6 Hz, 2H), 1.69–1.59 (m, 2H), 1.40 (dtt, *J* = 14.5, 7.3, 3.2 Hz, 1H), 1.15 (dqd, *J* = 24.7, 12.2, 3.7 Hz, 2H). ^13^C NMR (126 MHz, CD_3_OD) δ 143.14, 137.67, 133.70, 131.73, 130.36, 130.33, 130.03, 129.28, 127.36, 126.84, 126.60, 125.27, 113.19, 59.65, 58.14, 53.26, 52.97, 48.19, 48.15, 48.12, 48.06, 47.95, 47.89, 47.78, 47.72, 47.61, 47.44, 47.32, 47.27, 47.10, 35.94, 35.86, 29.45, 20.03. HRMS (ESI): calcd. For C_21_H_25_F_3_N_2_O_2_S [M + H]^+^ 427.1662, found 427.1660.

### 3.7. General Procedure for the Synthesis of ***16a**–**d***

Ethyl 2-piperidin-4-ylacetate **11** (1 g, 5.84 mmol, 1.0 equiv.) and substituted (2-romoethyl)benzene derivatives (**12a**–**b**) (7.01 mmol, 1.2 equiv.) were dissolved in 20 mL acetone. Then, anhydrous K_2_CO_3_ (11.68 mmol, 2 equiv.) and catalytic amount KI were added. The reaction mixture was refluxed for 4 h. After completion of the reaction, acetone was concentrated, and the residue was dissolved in water (60 mL) and extracted with ethyl acetate (60 × 3 mL). The combined organic layers were dried over Na_2_SO_4_, filtered, and concentrated in vacuum. The obtained oil was used in further synthesis without purification yielded compounds **13a**–**b** (Yields were 67% and 72%). Then, 4 mol/L KOH (2.5 equiv.) was added to the solution of compounds **13a**–**b** in C_2_H_5_OH: H_2_O = 5:1(6 mL). The reaction mixture was stirred at room temperature for 7 h. After completion of the reaction, the reaction mixture was evaporated to dryness after neutralization with dilute hydrochloric acid solution. Poured into ethyl acetate to deposit the solid, after cooling off, the mixture was filtered and washed with cold ethyl acetate to give compounds **14a**–**d**.

Finally, intermediates (**14a**–**b**) (1.2 equiv.), PyBOP (1.2 equiv.) and DIPEA (1.5 equiv.) were added to 6 mL DMF and stirred at room temperature for 20 min. Then, intermediates (**15a**–**b**) (1.0 equiv.) was added and stirred at room temperature for 4 h. After completion of the reaction, the reaction mixture was quenched with saturated NaCl solution. The aqueous phase was extracted with DCM. The DCM layer was combined and washed with brine solution. The organic layer was dried over anhydrous Na_2_SO_4_ and the solvent was removed under reduced pressure. After concentration, the crude product was purified by silica gel column chromatograph using a methanol in dichloromethane gradient (DCM:methanol = 60:1–5:1) yielded compounds **16a**–**d**.

*N-(1H-Indol-5-yl)-2-(1-phenethylpiperidin-4-yl)acetamide* (**16a**). 2-(1-Phenethylpiperidin-4-yl) acetic acid (140 mg, 0.57 mmol), 1*H*-Indol-5-amine (63 mg, 0.48 mmol), PyBOP (295 mg, 0.57 mmol), DIPEA (93 mg, 0.72 mmol), DMF (6 mL). Yellow solid, m.p.: 165–167 °C, yield: 80.70%, ^1^H NMR (400 MHz, CD_3_OD) δ 7.74 (d, *J* = 2.0 Hz, 1H), 7.28 (tt, *J* = 13.1, 6.8 Hz, 6H), 7.20 (d, *J* = 3.1 Hz, 1H), 7.15 (dd, *J* = 8.6, 2.0 Hz, 1H), 6.37 (d, *J* = 3.1 Hz, 1H), 3.60 (d, *J* = 14.9 Hz, 2H), 3.26 (d, *J* = 5.0 Hz, 1H), 3.08–3.01 (m, 3H), 2.39 (d, *J* = 7.0 Hz, 2H), 2.21–2.13 (m, 1H), 2.04 (d, *J* = 15.4 Hz, 2H), 1.74–1.60 (m, 2H), 1.27 (s, 2H). ^13^C NMR (151 MHz, CD_3_OD) δ 170.66, 136.38, 133.81, 129.79, 128.59, 128.42, 127.97, 126.86, 125.29, 115.83, 115.76, 112.47, 110.75, 101.07, 72.24, 70.09, 60.80, 57.69, 53.42, 52.37, 41.90, 31.68, 31.23, 29.38, 28.89, 22.34, 13.05. HRMS (ESI): calcd. For C_23_H_27_N_3_O [M + H]^+^ 362.2227, found 362.2242.

*2-(1-(2-Chlorophenethyl)piperidin-4-yl)-N-(1H-indol-5-yl)acetamide* (**16b**). 2-(1-(2-Chlorophenethyl)piperidin-4-yl)acetic acid (140 mg, 0.50 mmol), 1H-Indol-5-amine (55 mg, 0.42 mmol), PyBOP (260 mg, 0.50 mmol), DIPEA(80 mg, 0.62 mmol), DMF (6 mL). Yellow solid, m.p.: 172–173 °C, yield: 30.41%, ^1^H NMR (400 MHz, CD_3_OD) δ 7.75 (d, *J* = 1.9 Hz, 1H), 7.39–7.29 (m, 3H), 7.28–7.20 (m, 2H), 7.19 (d, *J* = 3.1 Hz, 1H), 7.16 (dd, *J* = 8.7, 2.0 Hz, 1H), 6.37 (d, *J* = 4.0 Hz, 1H), 3.45 (d, J = 12.3 Hz, 2H), 3.15–3.09 (m, 2H), 3.08–3.01 (m, 2H), 2.79 (t, J = 13.0 Hz, 2H), 2.36 (d, *J* = 7.1 Hz, 2H), 2.10 (s, 1H), 2.00–1.92 (m, 2H), 1.60 (q, *J* = 11.5 Hz, 2H). ^13^C NMR (151 MHz, CD_3_OD) δ 171.22, 135.56, 133.78, 133.57, 130.84, 129.85, 129.30, 128.28, 127.96, 127.17, 125.21, 115.80, 112.48, 110.73, 101.06, 56.91, 52.68, 42.55, 32.24, 29.95, 29.32, 29.01. HRMS (ESI): calcd. For C_23_H_26_ClN_3_O [M + H]^+^ 396.1837, found 396.1838.

*N-(1H-Benzo[d]imidazol-5-yl)-2-(1-phenethylpiperidin-4-yl)acetamide* (**16c**). 2-(1-phenethylpiperidin-4-yl)acetic acid (140 mg, 0.57 mmol), 1*H*-Benzo[*d*]imidazol-5-amine (64 mg, 0.48 mmol), PyBOP (295 mg, 0.57 mmol), DIPEA(93 mg, 0.72 mmol), DMF (6 mL). Red solid, m.p.: 167–168 °C, yield: 45.92%, ^1^H NMR (400 MHz, CD_3_OD) δ 8.09 (s, 1H), 8.03 (s, 1H), 7.52 (d, *J* = 8.7 Hz, 1H), 7.29–7.12 (m, 6H), 3.07 (d, *J* = 12.0 Hz, 2H), 2.84–2.78 (m, 2H), 2.64–2.58 (m, 2H), 2.32 (d, *J* = 7.2 Hz, 2H), 2.17 (t, *J* = 11.8 Hz, 2H), 1.93 (s, 1H), 1.81 (d, *J* = 12.9 Hz, 2H), 1.42 (q, *J* = 12.1, 10.5 Hz, 2H). ^13^C NMR (101 MHz, CD_3_OD) δ 171.84, 141.67, 139.67, 133.93, 128.33, 128.21, 128.20, 125.93, 116.32, 60.33, 53.19, 47.89, 43.30, 33.27, 32.51, 31.15. HRMS (ESI): calcd. For C_22_H_26_N_4_O [M + H]^+^ 363.2179, found 363.2197.

*N-(1H-Benzo[d]imidazol-5-yl)-2-(1-(2-chlorophenethyl)piperidin-4-yl)acetamide* (**16d**). 2-(1-(2-Chlorophenethyl)piperidin-4-yl)acetic acid (140 mg, 0.50 mmol), 1*H*-Benzo[*d*]imidazol-5-amine (55 mg, 0.42 mmol), PyBOP (259 mg, 0.50 mmol), DIPEA(80 mg, 0.62 mmol), DMF (6 mL). Red solid, m.p.: 176–177 °C, yield: 40.33%, 1H NMR (600 MHz, CDCl_3_) δ 7.95 (s, 1H), 7.70 (s, 1H), 7.66 (d, *J* = 8.1 Hz, 1H), 7.48 (d, *J* = 7.4 Hz, 1H), 7.35 (d, *J* = 7.8 Hz, 1H), 7.24 (t, *J* = 7.4 Hz, 1H), 7.19 (t, *J* = 7.3 Hz, 1H), 6.90 (d, *J* = 8.1 Hz, 1H), 3.62 (s, 2H), 3.57 (d, *J* = 11.7 Hz, 1H), 3.37 (d, *J* = 6.5 Hz, 2H), 2.95 (d, *J* = 11.2 Hz, 2H), 2.10 (t, *J* = 11.3 Hz, 2H), 1.75 (d, *J* = 12.4 Hz, 2H), 1.66 (s, 1H), 1.60 (s, 1H), 1.39 (q, *J* = 11.5 Hz, 2H). ^13^C NMR (151 MHz, CD_3_OD) δ 171.90, 141.65, 137.34, 134.98, 133.95, 133.58, 130.73, 129.20, 127.73, 126.97, 121.27, 116.28, 58.31, 58.27, 53.17, 53.06, 50.62, 43.39, 40.15, 33.39, 32.59, 31.29, 31.15, 30.24. HRMS (ESI): calcd. For C_22_H_25_ClN_4_O [M + H]^+^ 397.1790, found 397.1797.

### 3.8. AChE and BChE Inhibition Assay

The inhibitory activity of the target compound against AChE (from Electrophorus electricus (eeAChE), Sigma-Aldrich, Munich, Germany) and horse serum BChE (eqBChE, Sigma-Aldrich, Munich, Germany) were measured by Ellman’s method [32]. In 96-well plates, a mixture of phosphate buffer (0.1 M, pH 8.0, 2 mL), 5,5′-dithiobis-2-nitrobenzoic acid (DTNB, 60 μL), acetylcholinesterase or butyrylcholinesterase (20 μL, 5 IU/mL) and different concentrations of the compounds solution (30 μL) was pre-incubated for 5 min and then substrates (acetylthiocholine iodide or butyrylthiocholine iodide, 20 μL) were added.

Changes in absorbance were measured at 412 nm by using microplate reader (Thermo, Varioskan Flash 3001, Thermo Fisher Scientific, Agawam, MA, USA). The measurement of each concentration for each compound was detected in triplicate. GraphPad Prism 6.0 (GraphPad Software, Inc., La Jolla, CA, USA) was used for data processing. The inhibition curve was fitted by plotting the logarithm of the concentration of the tested compounds with the percentage of enzyme activity (reference to 100%). The (IC_50_) value was calculated according to the inhibition curve and the data were shown in the layout of mean ± SEM by GraphPad Prism 6.0.

### 3.9. Kinetics of AChE and BChE Inhibition

Kinetic studies were performed in the same manner as the determination of ChEs inhibition, with substrate (ATC/BTC) concentrations of 90, 150, 226, 452 and 904 μM. The concentration of compound **8i** was set to 0, 0.25, 0.5, 0.75, 1 μM, and the concentration of compound **10s** was set to 1, 2.5, 5, 7.5, 10 μM. The enzymatic reaction was extended to 7 min for eeAChE and eqBChE before the determination of the absorption. The Vmax and Km values of Michaelis-Menten kinetics were calculated by nonlinear regression from the substrate-velocity curves using GraphPad Prism 6.0. Linear regression was used to fit the Lineweaver–Burk plot.

### 3.10. Molecular Docking Study

The crystal structures of eeAChE (PDB:1C2B) [36], huAChE (PDB ID: 4EY7) [32] and huBChE (PDB ID: 4TPK) [37] were obtained from the Research Collaboratory for Structural Bioinformatics Protein Data Bank (RCSB PDB). The Discovery Studio software 2016 (DS 2016, BIOVIA, San Diego, CA, USA) was used to study the docking of compound **8i.** The three protein structures are pretreated (i.e., protonated, removed water, added Miss sidechains, etc.) by the “prepare protein” module in DS to provide the structures suitable for docking. The “prepare ligand” module in DS is used to test the structural preparation of the compound. The native ligand in the crystal structure was used to define the binding site. The binding site was defined as the site sphere ((in 10 Å radius) around the original ligand in the co-crystal structures. The docking program CDOCKER encoded in DS 2016 was applied to identify the potential binding of compound **8i** to eeAChE, huAChE, and huBChE. Other CDOCKER parameters were set to default values. Compound **8i** was chosen for molecular modeling as the most active compounds in the series (Table 1). Compound **8i** produced 10 poses to eeAChE, huAChE, and huBChE. These postures were visually examined, and the most appropriate docking pose was selected according to the scores and interactions with key residues of the eeAChE, huAChE, and huBChE active sites.

### 3.11. Cell Studies in Vitro

Pheochromocytoma-derived cell line (PC12 cells) were grown in Dulbecco’s Modified Eagle Medium (DMEM) supplemented with 10% FBS at 37 °C in a humidified atmosphere containing 5% CO_2_. To carry out the experiment, cells (6 × 10^3^ cells/well) were seeded in 96-well plate in complete medium. After 24 h, the culture medium was removed and the cells were exposed to increasing concentrations of compounds **6b**, **6c**, **6d**, **8i**, **10s**, **10v** or Tacrine (10, 20, 30 and 50 μM) in DMEM for further 24 h. Cell survival was measured by 3-(4,5-Dimethylthiazol-2-yl)-2,5-Diphenyltetrazolium Bromide (MTT) assay [37].

## 4. Conclusions

In this paper, a series of novel compounds **6a**–**h**, **8i**–**1**, **10s**–**v**, and **16a**–**d** were synthesized and evaluated, together with the known analogs **11a**–**f**, for their inhibitory activities towards AChE and BChE. The results show that most of the compounds have AChE and/or BChE inhibitory activity. Compound **8i** showed the strongest inhibitory effect on both AChE (eeAChE IC_50_ = 0.39 μM) and BChE (eqBChE IC_50_ = 0.28 μM). Compared with compound **G801-0274**, compound **8i** has comparable inhibitory activity against two ChEs, so that it can exert an anti-ChEs effect in a balanced manner. Kinetic studies indicated a mixed-type inhibition of compound **8i**, including competitive inhibition and non-competitive inhibition. Subsequently, molecular docking was performed to evaluate the interaction mechanism between compound **8i** and enzymes. Enzyme inhibition kinetics and molecular modeling studies have shown that compound **8i** bind simultaneously to the PAS and the CAS of AChE and BChE. Therefore, compounds **8i** may be promising scaffold for treatment, and further modifications have been made to obtain novel AChE and BChE dual-target inhibitors.

## Figures and Tables

**Table 1:** Structures, eeAChE, and eqBChE inhibitory activities of target compounds.

Compound	Structure	AChE^a^ (IC_50_^c^, μM or IR^d^, %)	BChE ^b^ (IC_50_, μM or IR ^d^, %)	SI ^e^
**6a**	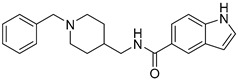	52.11 ± 30.25	31.37 ± 15.56	1.66
**11a ^f^**	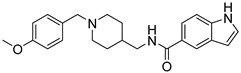	38.08 ± 12.83	10.75 ± 5.55	3.54
**6b**	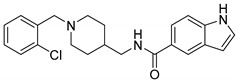	15.02 ± 6.17	9.56 ± 4.52	1.57
**6c**	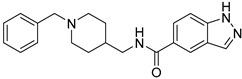	9.06 ± 2.57	36.64 ± 17.54	0.25
**11b ^f^**	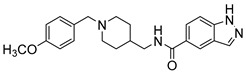	48.57 %	44.57 %	--
**11c ^f^**	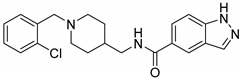	5.44 ± 2.22	21.29 ± 5.00	0.26
**6d**	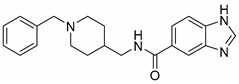	4.80 ± 0.68	2.15 ± 0.63	2.23
**6e**	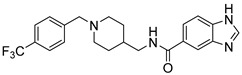	48.48 ± 36.19	39.88%	--
**11d ^f^**	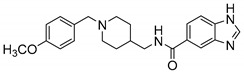	18.81 %	16.51 %	--
**11e ^f^**	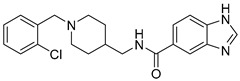	7.48 ± 3.10	42.12 %	--
**11f ^f^**	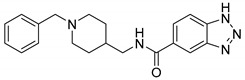	0.46 ± 0.40	43.07 %	--
**6f**	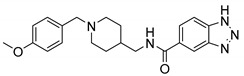	43.07 %	49.63 %	--
**6g**	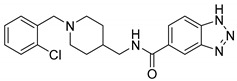	29.20 ± 16.38	49.87 ± 37.29	0.58
**6h**	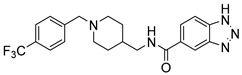	40.20 ± 27.64	30.71%	--
**8i**	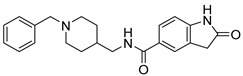	0.39 ± 0.04	0.28 ± 0.10	1.39
**8k**	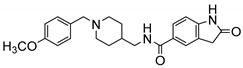	no	22.26%	--
**8j**	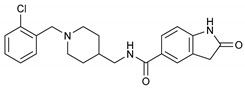	14.69%	22.87%	--
**8l**	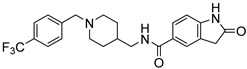	21.54 ± 9.37	35.20%	--
**10s**	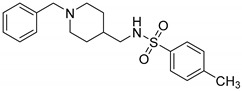	4.24 ± 2.44	4.10 ± 3.74	1.03
**10t**	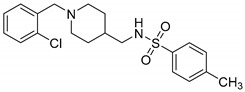	5.50 ± 2.56	2.01 ± 0.70	2.74
**10u**	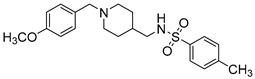	11.61%	18.31%	--
**10v**	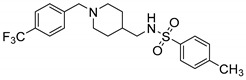	7.20 ± 10.75	7.14 ± 5.27	1.01
**16a**	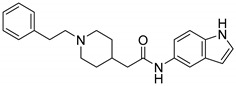	8.61%	23.49%	--
**16b**	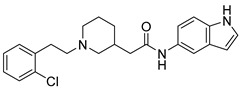	8.95%	31.21%	--
**16c**	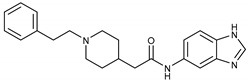	no	30.59%	--
**16d**	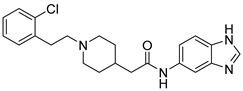	8.55%	38.04%	--
**Tacrine**	_	0.02 ± 0.01	0.008 ± 0.004	2.50
**Donepezil**	_	0.008 ± 0.002	1.734 ± 0.731	0.0046

^a^ AChE (EC 3.1.1.7) from electric eel. ^b^ BChE (EC 3.1.1.8) from horse serum. ^c^ Concentration required for 50% inhibition of ChEs, data were shown in mean ± SEM of triplicate independent experiments. ^d^ Inhibitory rate of the compounds under 100 μM on ChEs. ^e^ Selectivity index (SI) = AChE IC_50_/BChE IC_50._^f^ The known analogs **11a**–**f** [33].

## Supplementary Materials

Copies of the ^1^H-NMR and ^13^C-NMR spectra of the compounds are available online.
