# Supplementary material for: Design, Synthesis, and Evaluation of Acetylcholinesterase and Butyrylcholinesterase Dual-Target Inhibitors against Alzheimer’s Diseases
Source: Molecules. 2020 Jan 23;25(3):489. doi: 10.3390/molecules25030489 (PMC7038160; doi:10.3390/molecules25030489)

**Supporting Information**

# Design, synthesis and evaluation of acetylcholinesterase and butyrylcholinesterase dual-target inhibitors against Alzheimer's disease

**Yan Guo [1], Hongyu Yang [2], Zhongwei Huang [1], Sen Tian [1], Qihang Li [2], Chenxi Du [2], Tingkai Chen [3], Yang Liu [2], Haopeng Sun [2,4,*] and Zongliang Liu [1,*]**

[1] School of Pharmacy, Key Laboratory of Molecular Pharmacology and Drug Evaluation (Yantai University), Ministry of Education, Collaborative Innovation Center of Advanced Drug Delivery System and Biotech Drugs in Universities of Shandong, Yantai University, Yantai, 264005, P.R. China; 18865672173@163.com (Y.G.); 15809573905@163.com (Z.H.); 18863665626@163.com (S.T.)

[2] School of Pharmacy, China Pharmaceutical University, Nanjing, 211198, People's Republic of China; yhykjy@sina.com (H.Y.); liqihangcpu@163.com (Q.L.); 15651670339@163.com (C.D.); lyliuyang1997@163.com (Y.L.)

[3] School of Traditional Chinese Pharmacy, China Pharmaceutical University, Nanjing, 211198, People's Republic of China; 18013972875@163.com (T.C.)

[4] Jiangsu Food and Pharmaceutical Science College, Huaian, 223003, People's Republic of China

* Correspondence: sunhaopeng@163.com (H.S.); lzl_0_0@126.com (Z.L.); Tel.: +86-0535-6706023 (Z.L.)

Received: 24 December 2019; Accepted: 19 January 2020; Published: 23 January 2020

**The $^{1}$H NMR, $^{13}$C NMR and HRMS (ESI) spectrum of target compounds**

**6a**

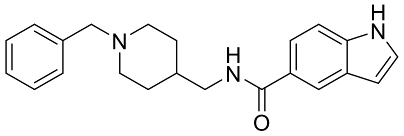

$^{1}$H NMR

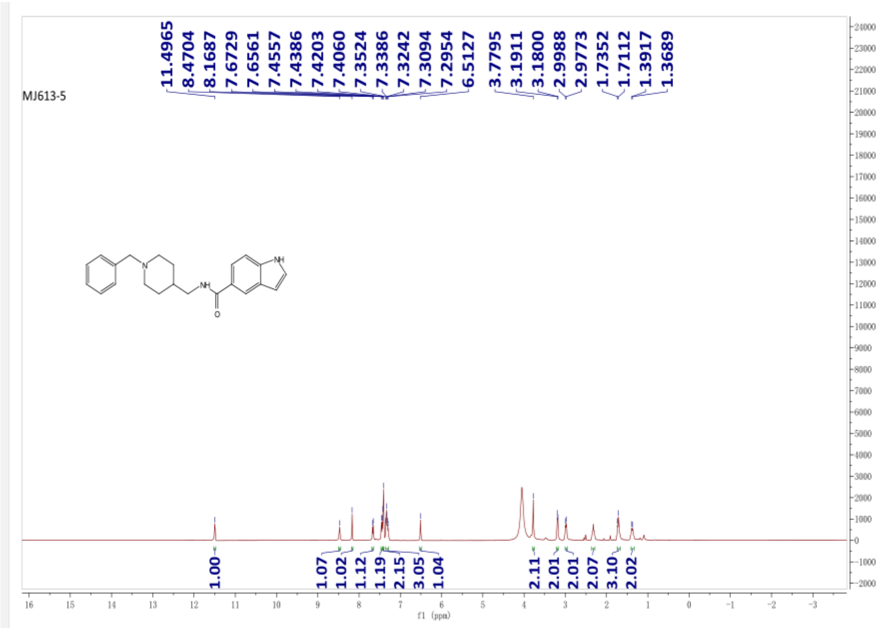

$^{1}$H NMR (500 MHz, DMSO-$d_6$) δ 11.50 (s, 1H), 8.47 (s, 1H), 8.17 (s, 1H), 7.66 (d, *J* = 8.4 Hz, 1H), 7.45 (d, *J* = 8.6 Hz, 1H), 7.41 (d, *J* = 7.1 Hz, 2H), 7.36 – 7.29 (m, 3H), 6.51 (s, 1H), 3.78 (s, 2H), 3.19 (d, *J* = 5.5 Hz, 2H), 2.99 (d, *J* = 10.8 Hz, 2H), 2.31 (d, *J* = 11.0 Hz, 2H), 1.72 (d, *J* = 12.0 Hz, 3H), 1.38 (d, *J* = 11.4 Hz, 2H).

$^{13}$C NMR

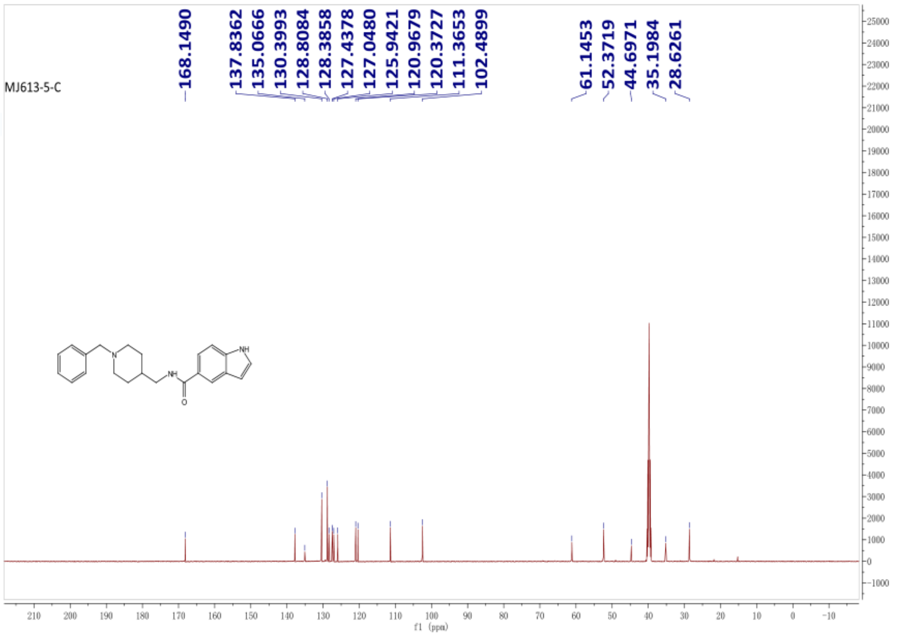

$^{13}C$ NMR (126 MHz, DMSO-$d_6$) δ 168.15, 137.84, 135.07, 130.40, 128.81, 128.39, 127.44, 127.05, 125.94, 120.97, 120.37, 111.37, 102.49, 61.15, 52.37, 44.70, 35.20, 28.63.

HRMS (ESI)

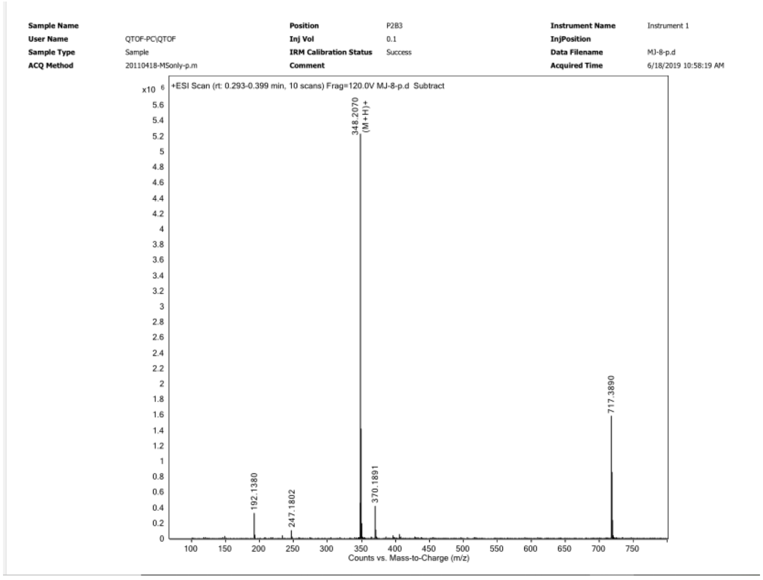


**6b**

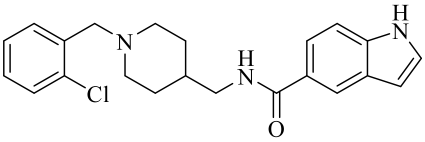

$^{1}H$ NMR

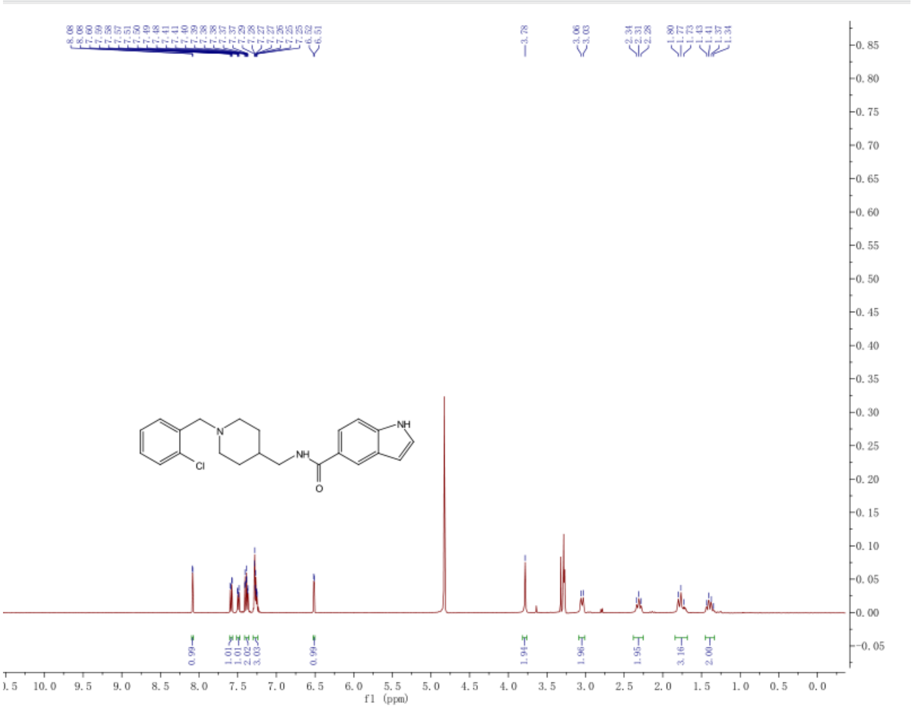


$^{1}H$ NMR (400 MHz, $CD_3OD$) δ 8.08 (d, *J* = 1.1 Hz, 1H), 7.58 (dd, *J* = 8.6, 1.8 Hz, 1H), 7.49 (dd, *J* = 7.1, 2.2 Hz, 1H), 7.41 – 7.35 (m, 2H), 7.30 – 7.24 (m, 3H), 6.51 (d,

*J* = 3.2 Hz, 1H), 3.78 (s, 2H), 3.05 (d, *J* = 11.7 Hz, 2H), 2.31 (t, *J* = 11.9 Hz, 2H), 1.77 (t, *J* = 14.0 Hz, 3H), 1.45 – 1.33 (m, 2H).

$^{13}$C NMR

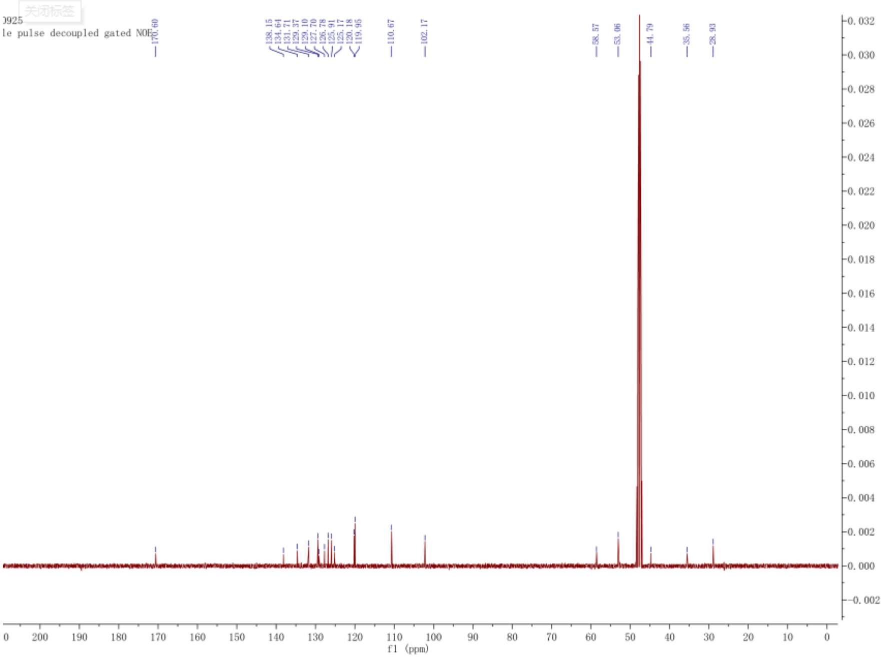

$^{13}$C NMR (101 MHz, $CD_3OD$) δ 170.60, 138.15, 134.64, 131.71, 129.37, 129.10, 127.70, 126.78, 125.91, 125.17, 120.18, 119.95, 110.67, 102.17, 58.57, 53.06, 44.79, 35.56, 28.93.

HRMS (ESI)

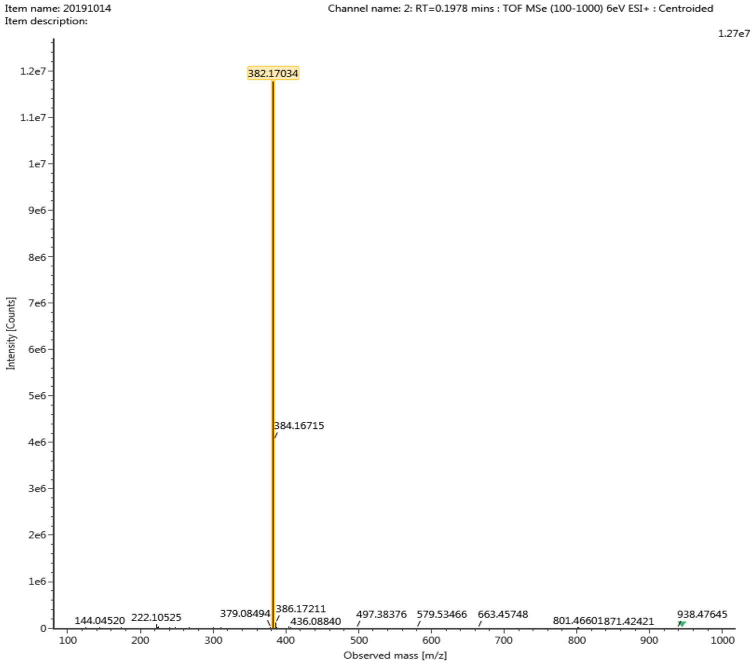


**6c**

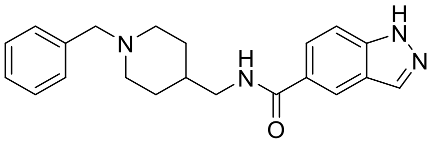

$^{1}$H NMR

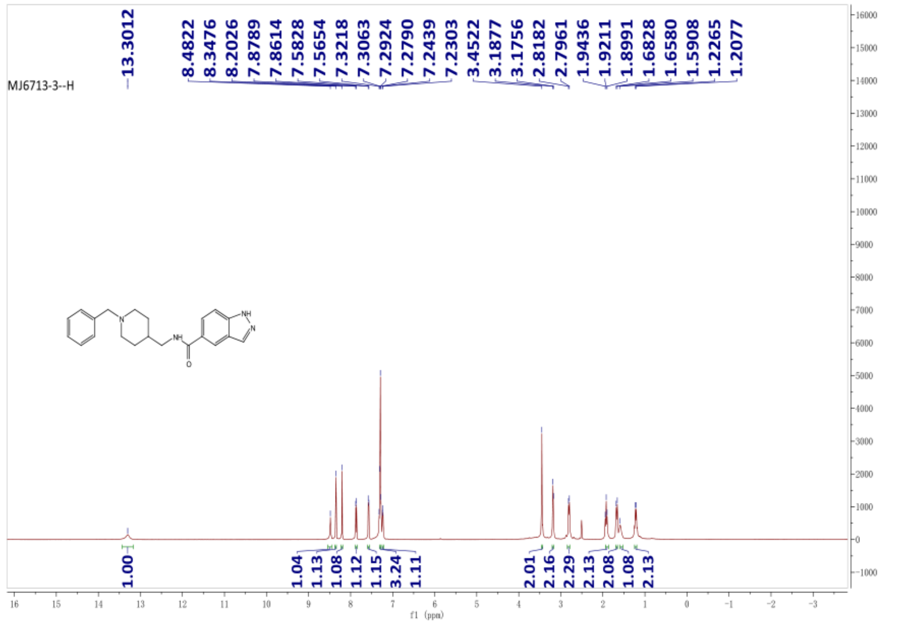


$^{1}$H NMR (500 MHz, DMSO-$d_6$) δ 13.30 (s, 1H), 8.48 (s, 1H), 8.35 (s, 1H), 8.20 (s, 1H), 7.87 (d, *J* = 8.8 Hz, 1H), 7.57 (d, *J* = 8.7 Hz, 1H), 7.33 – 7.27 (m, 3H), 7.24 (d, *J* = 6.8 Hz, 1H), 3.45 (s, 2H), 3.18 (d, *J* = 6.0 Hz, 2H), 2.81 (d, *J* = 11.1 Hz, 2H), 1.92 (t, *J* = 11.1 Hz, 2H), 1.67 (d, *J* = 12.4 Hz, 2H), 1.59 (s, 1H), 1.22 (d, *J* = 9.4 Hz, 2H).

$^{13}$C NMR

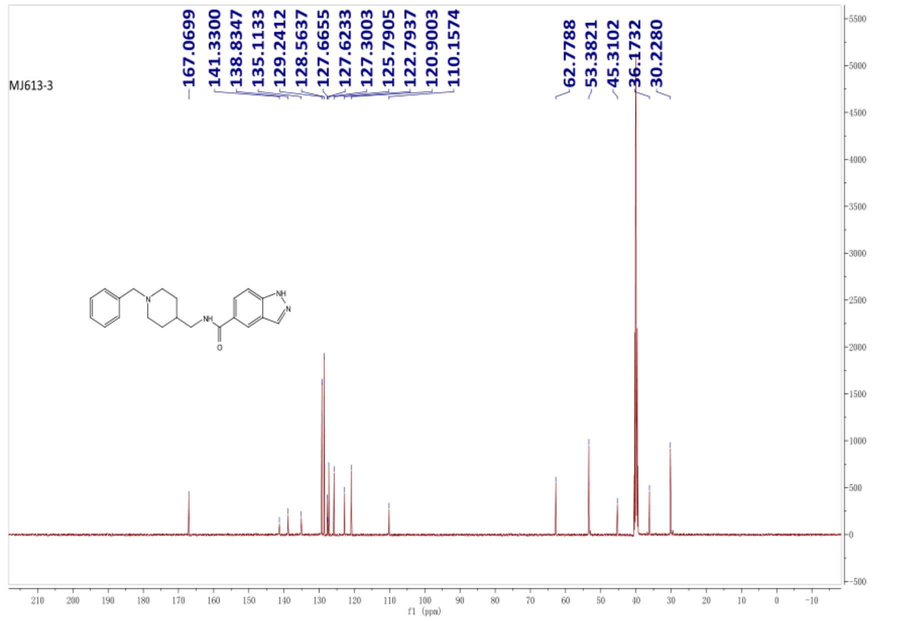


$^{13}$C NMR (126 MHz, DMSO-$d_6$) δ 167.07, 141.33, 138.83, 135.11, 129.24, 128.56, 127.67, 127.62, 127.30, 125.79, 122.79, 120.90, 110.16, 62.78, 53.38, 45.31, 36.17, 30.23.

HRMS (ESI)

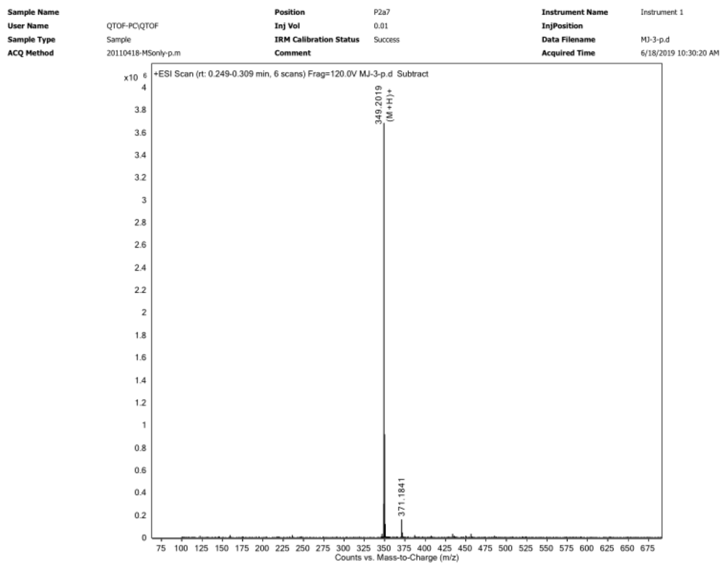


**6d**

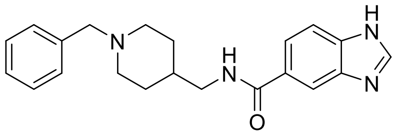

[1] H NMR

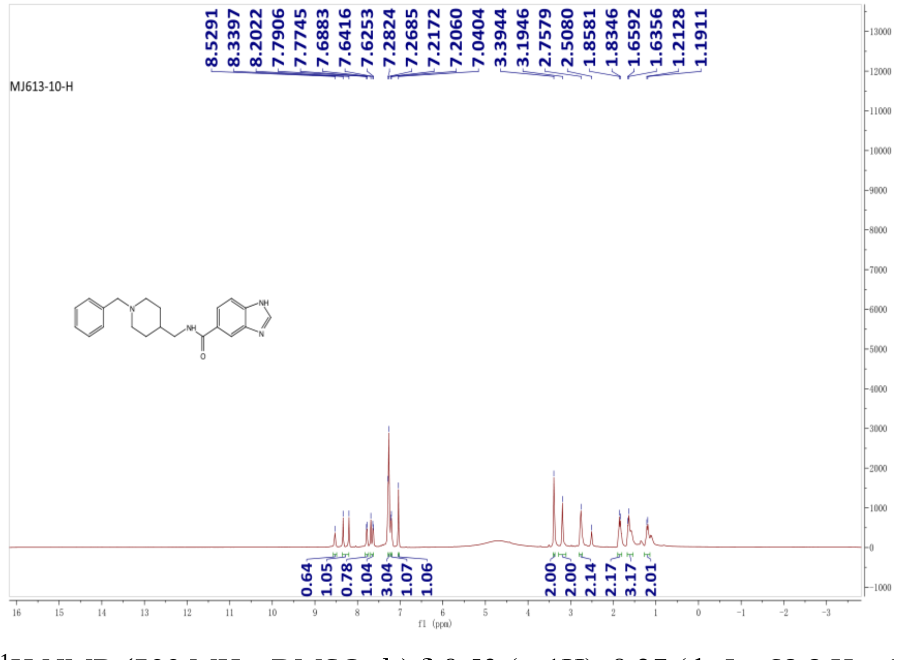


$^{1}$H NMR (500 MHz, DMSO-d$_6$) δ 8.53 (s, 1H), 8.27 (d, *J* = 68.8 Hz, 1H), 7.78 (d, *J* = 8.0 Hz, 1H), 7.70 – 7.61 (m, 1H), 7.28 (d, *J* = 6.9 Hz, 3H), 7.21 (d, *J* = 5.6 Hz, 1H), 7.04 (s, 1H), 3.39 (s, 2H), 3.19 (s, 2H), 2.76 (s, 2H), 1.85 (d, *J* = 11.8 Hz, 2H), 1.65 (d, *J* = 11.8 Hz, 3H), 1.20 (d, *J* = 10.9 Hz, 2H).

$^{13}$C NMR

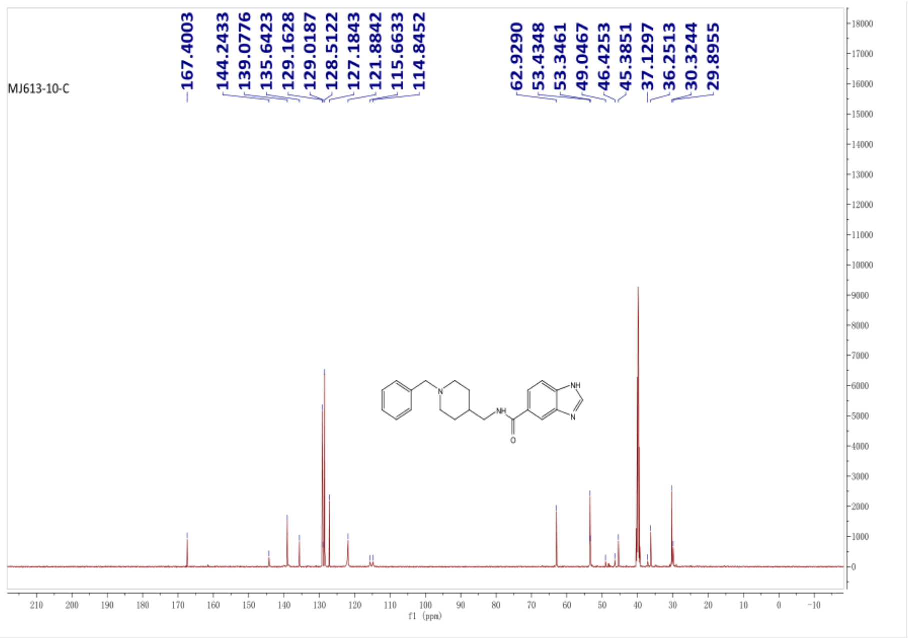


$^{13}C$ NMR (126 MHz, DMSO-$d_6$) δ 167.40, 144.24, 139.08, 135.64, 129.16, 129.02, 128.51, 127.18, 121.88, 115.66, 114.85, 62.93, 53.43, 53.35, 49.05, 46.43, 45.39, 37.13, 36.25, 30.32, 29.90.

HRMS (ESI)

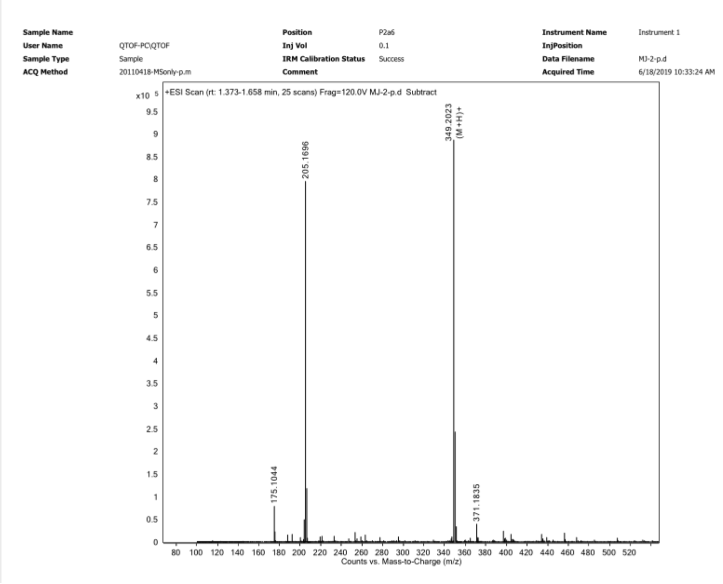


**6e**

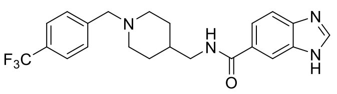

$^{1}$H NMR

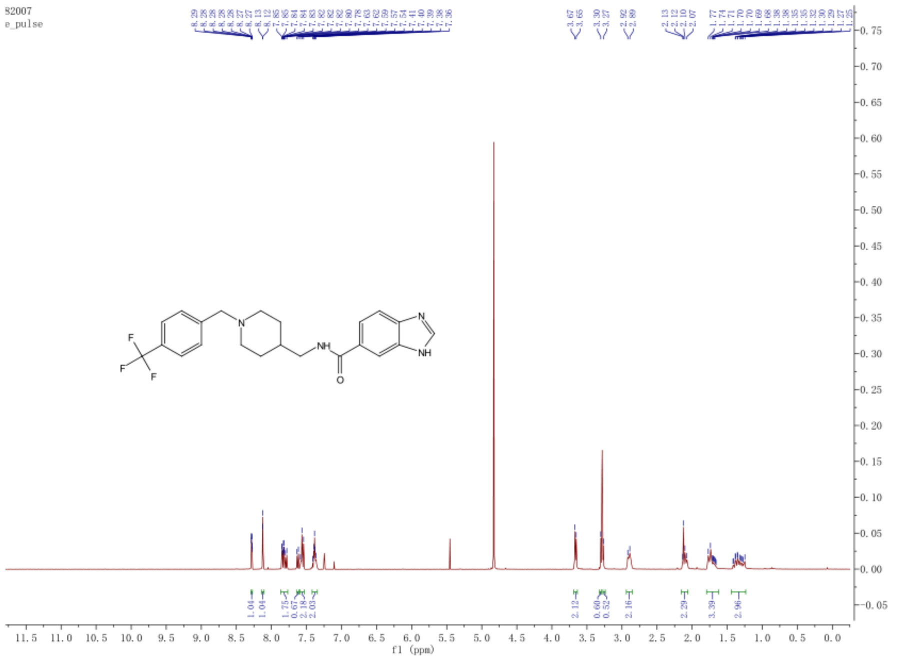

$^{1}$H NMR (400 MHz, $CD_3OD$) δ 8.29 – 8.27 (m, 1H), 8.12 (d, *J* = 1.0 Hz, 1H), 7.87 – 7.77 (m, 2H), 7.62 (d, *J* = 7.8 Hz, 1H), 7.56 (t, *J* = 8.9 Hz, 2H), 7.39 (p, *J* = 5.5 Hz, 2H), 3.66 (d, *J* = 6.7 Hz, 2H), 3.30 (s, 1H), 3.27 (s, 1H), 2.90 (d, *J* = 11.4 Hz, 2H), 2.15 – 2.06 (m, 2H), 1.79 – 1.63 (m, 3H), 1.44 – 1.24 (m, 3H).

$^{13}$C NMR

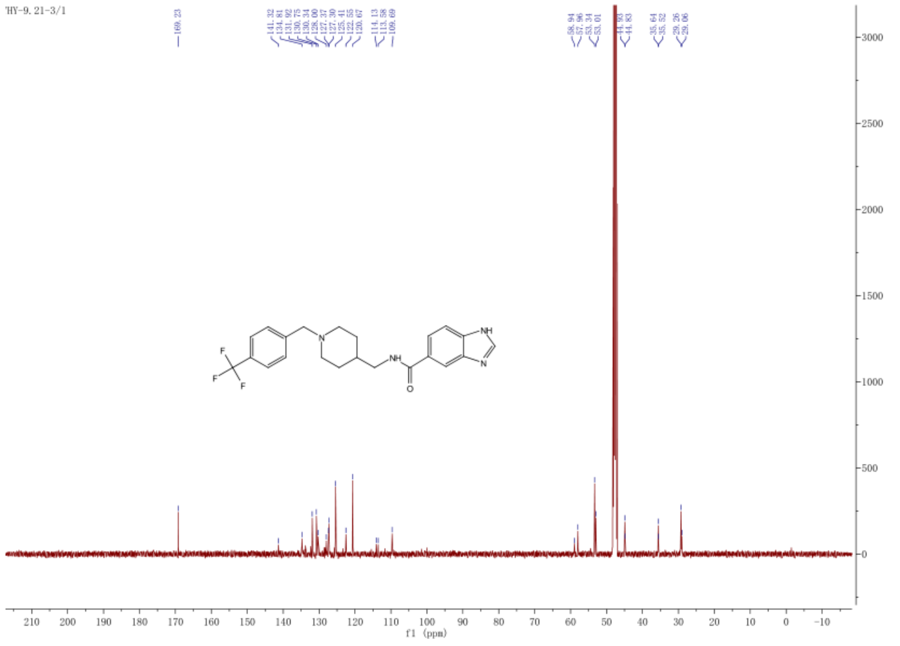

$^{13}$C NMR (126 MHz, $CD_3OD$) δ 169.23, 141.32, 134.81, 131.92, 130.75, 130.34, 128.00, 127.37, 127.30, 125.41, 122.55, 120.67, 114.13, 113.58, 109.69, 58.94, 57.96, 53.34, 53.01, 44.93, 44.83, 35.64, 35.52, 29.26, 29.06.

HRMS (ESI)

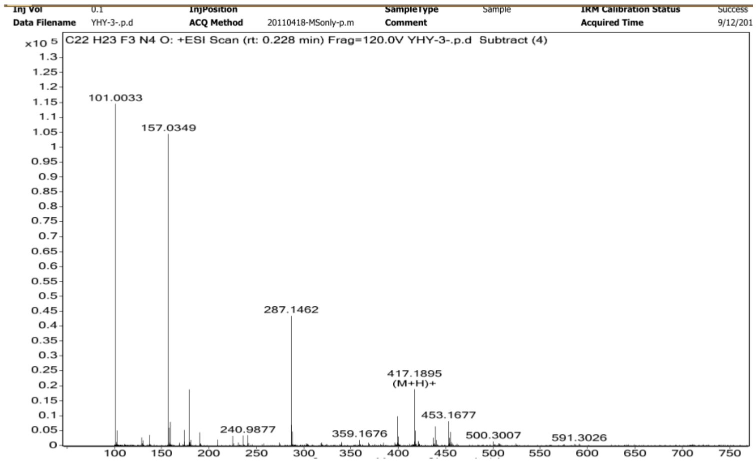


**6f**

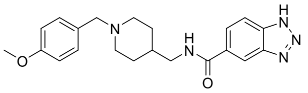

$^{1}$H NMR

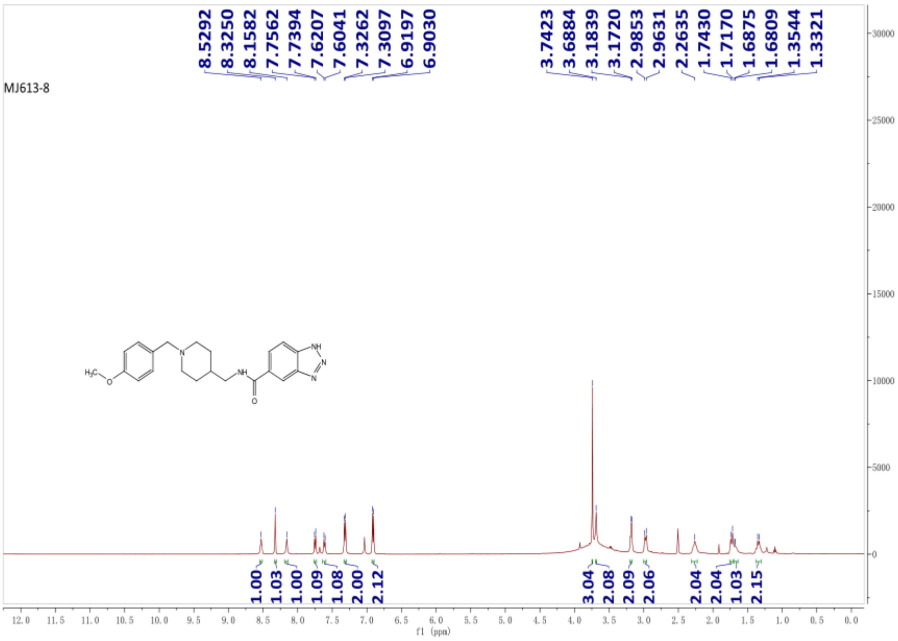


$^{1}$H NMR (500 MHz, DMSO-$d_6$) δ 8.53 (s, 1H), 8.32 (s, 1H), 8.16 (s, 1H), 7.75 (d, *J* = 8.4 Hz, 1H), 7.61 (d, *J* = 8.3 Hz, 1H), 7.32 (d, *J* = 8.3 Hz, 2H), 6.91 (d, *J* = 8.3 Hz, 2H), 3.74 (s, 3H), 3.69 (s, 2H), 3.18 (d, *J* = 5.9 Hz, 2H), 2.97 (d, *J* = 11.1 Hz, 2H), 2.26 (s, 2H), 1.73 (d, *J* = 13.0 Hz, 2H), 1.68 (s, 1H), 1.34 (d, *J* = 11.2 Hz, 2H).

$^{13}$C NMR

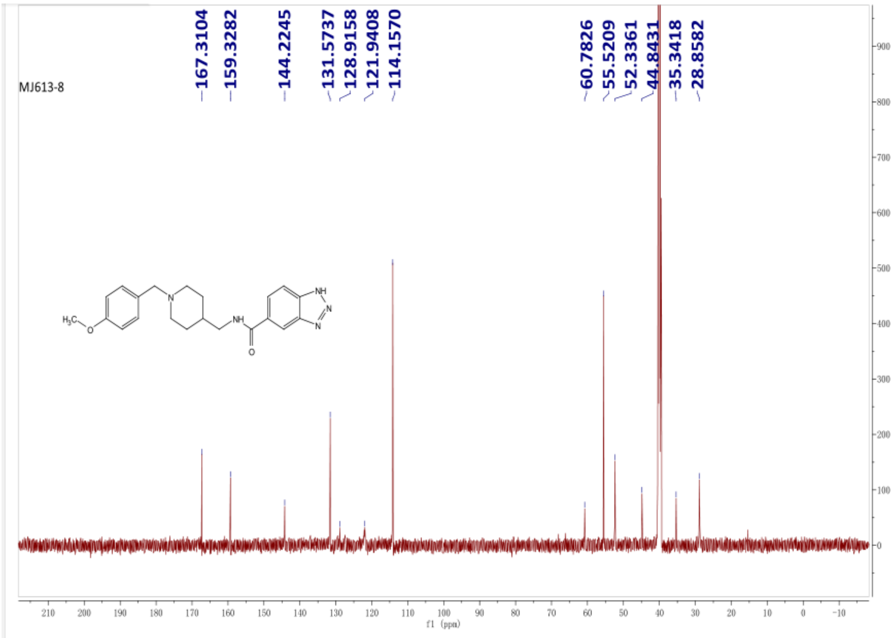


$^{13}$C NMR (126 MHz, DMSO-$d_6$) δ 167.31, 159.33, 144.22, 131.57, 128.92, 121.94, 114.16, 60.78, 55.52, 52.34, 44.84, 35.34, 28.86.

HRMS (ESI)

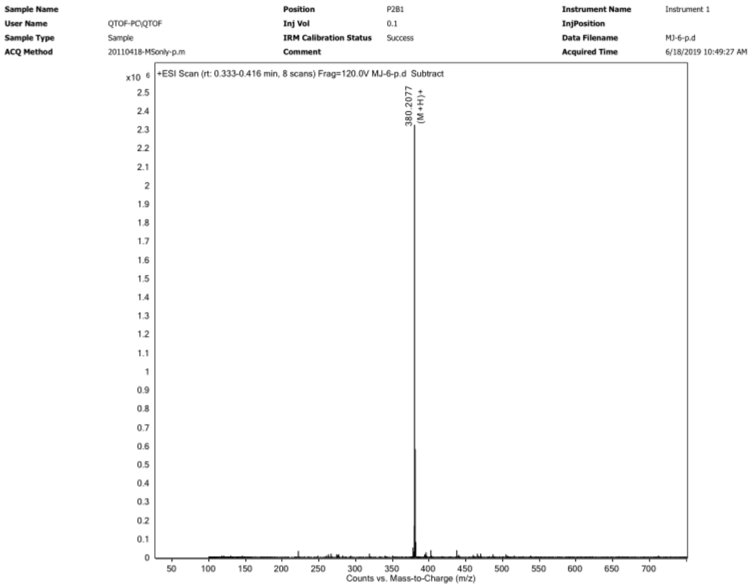


**6g**

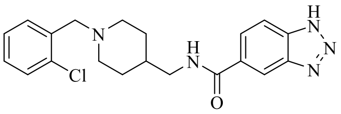

[1] H NMR

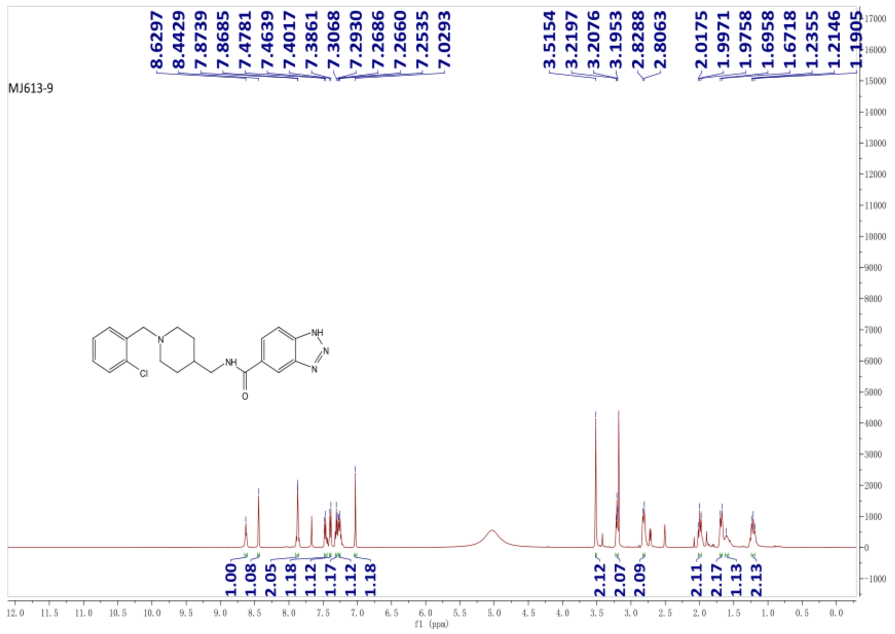


$^{1}$H NMR (500 MHz, DMSO-d$_6$) δ 8.63 (s, 1H), 8.44 (s, 1H), 7.87 (d, *J* = 2.7 Hz, 2H), 7.47 (d, *J* = 7.1 Hz, 1H), 7.39 (d, *J* = 7.8 Hz, 1H), 7.30 (d, *J* = 6.9 Hz, 1H), 7.27 – 7.25 (m, 1H), 7.03 (s, 1H), 3.52 (s, 2H), 3.21 (t, *J* = 6.1 Hz, 2H), 2.82 (d, *J* = 11.3 Hz, 2H), 2.00 (t, *J* = 10.4 Hz, 2H), 1.68 (d, *J* = 12.0 Hz, 2H), 1.61 (s, 1H), 1.23 (d, *J* = 10.5 Hz, 2H).

$^{13}$C NMR

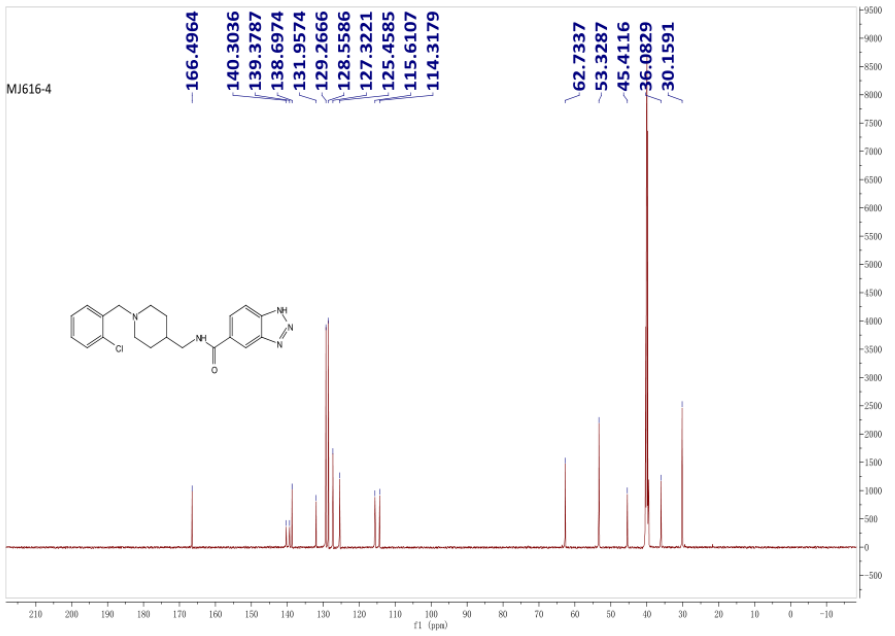


$^{13}$C NMR (126 MHz, DMSO-d$_6$) δ 166.50, 140.30, 139.38, 138.70, 131.96, 129.27, 128.56, 127.32, 125.46, 115.61, 114.32, 62.73, 53.33, 45.41, 36.08, 30.16.

HRMS (ESI)

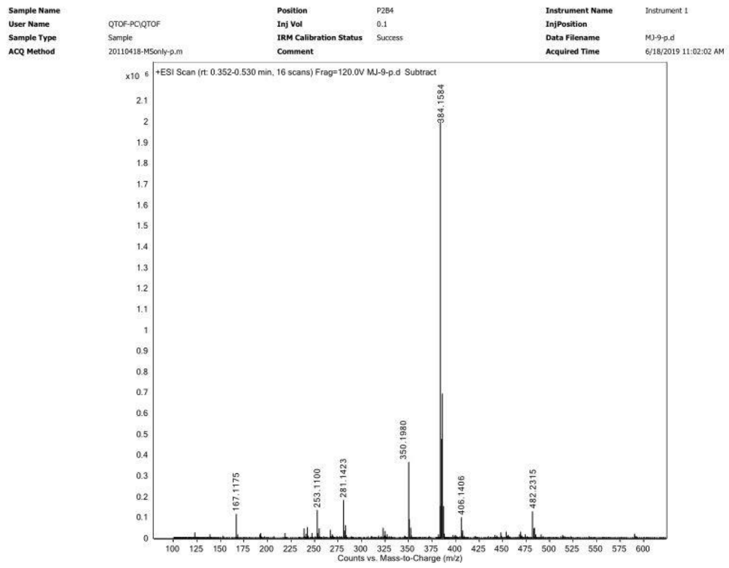

**6h**

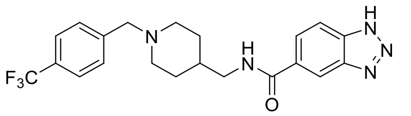

$^{1}$H NMR

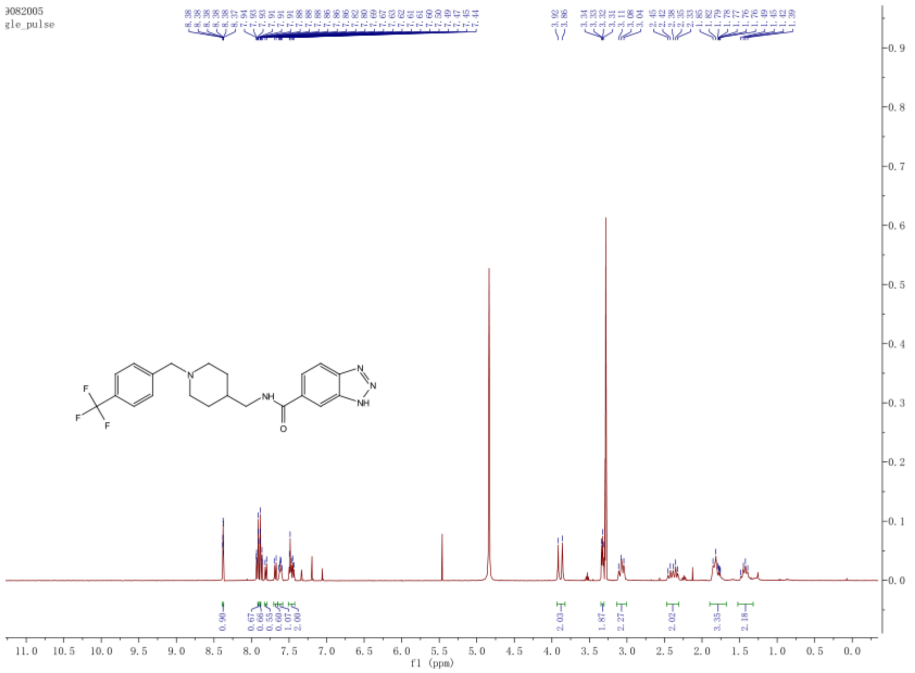

$^{1}$H NMR (400 MHz, $CD_3OD$) δ 8.38 (td, *J* = 1.5, 1.0 Hz, 1H), 7.92 – 7.90 (m, 1H), 7.88 (t, *J* = 1.0 Hz, 1H), 7.81 (d, *J* = 7.8 Hz, 1H), 7.68 (d, *J* = 7.8 Hz, 1H), 7.61 (dt, *J* = 7.7, 4.1 Hz, 1H), 7.51 – 7.42 (m, 2H), 3.89 (d, *J* = 22.6 Hz, 2H), 3.33 (dd, *J* = 6.5, 3.7 Hz, 2H), 3.13 – 3.01 (m, 2H), 2.47 – 2.31 (m, 2H), 1.90 – 1.68 (m, 3H), 1.52 – 1.32 (m, 2H).

$^{13}$C NMR

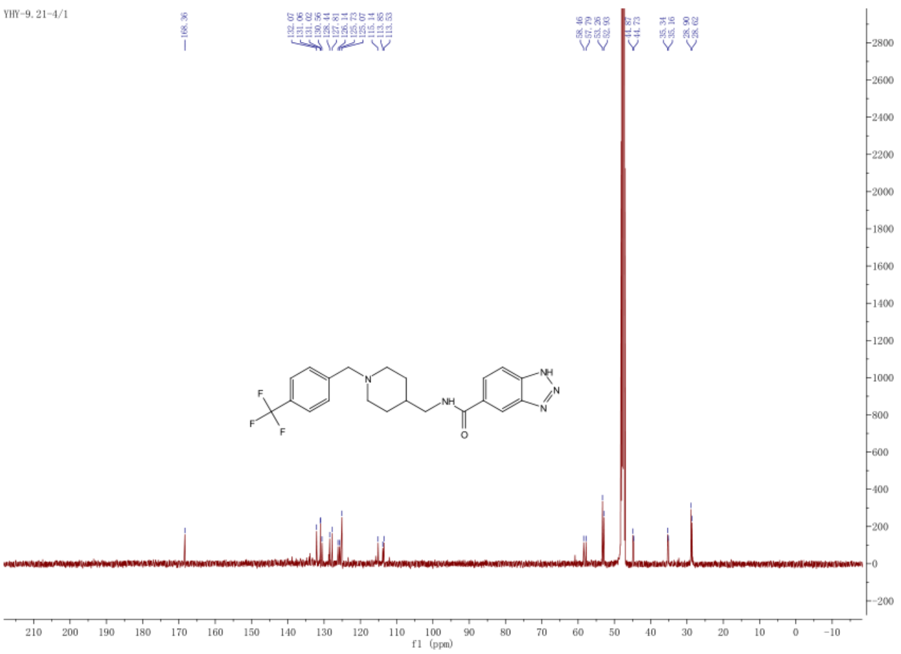

$^{13}C$ NMR (126 MHz, $CD_3OD$) δ 168.36 , 132.07 , 131.06 , 131.02 , 130.56 , 128.44 , 127.81 , 125.07 , 115.14 , 113.53 , 58.46 , 57.79 , 53.26 , 52.93 , 44.87 , 44.73 , 35.34 , 35.16 , 28.90 , 28.62 .

HRMS (ESI)

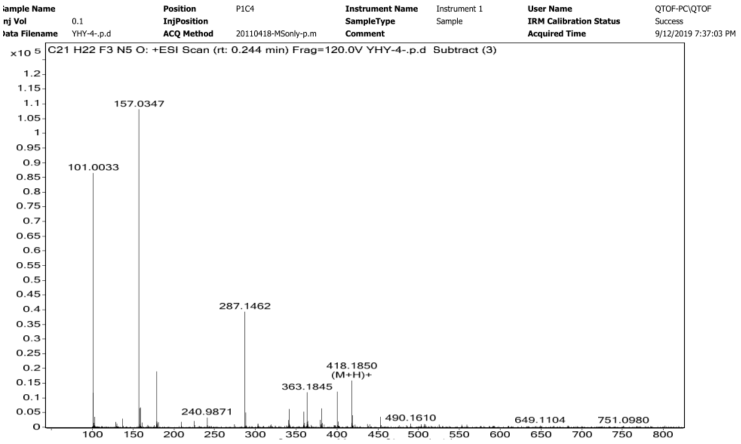


**8i**

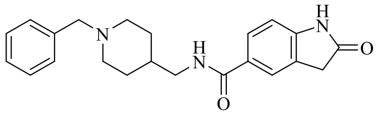

$^{1}H$ NMR

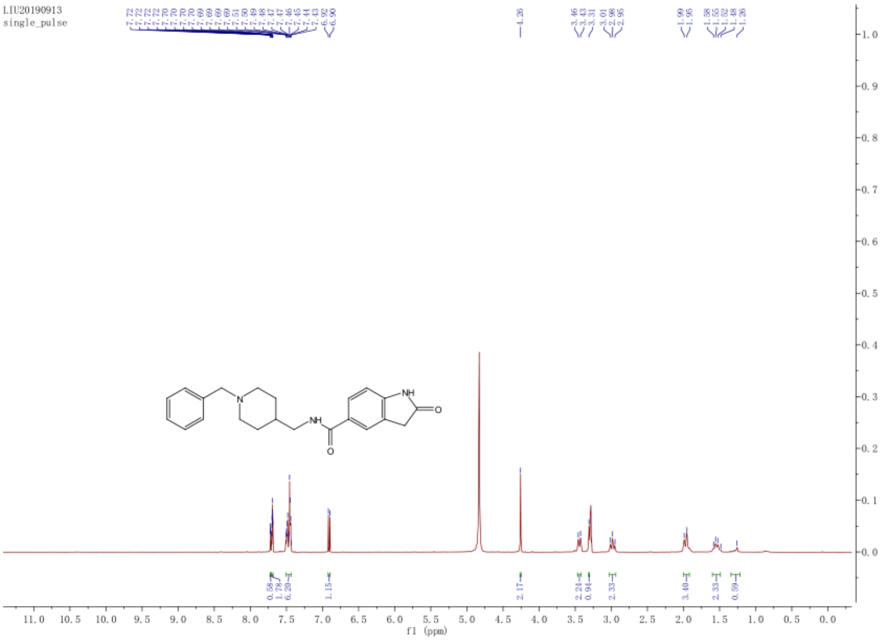

$^{1}$H NMR (400 MHz,$CD_3OD$) δ 7.72 (dd, *J* = 1.9, 0.4 Hz, 1H), 7.70 (ddd, *J* = 3.7, 1.5, 0.5 Hz, 2H), 7.47 (dq, *J* = 12.9, 3.6 Hz, 6H), 6.91 (d, *J* = 8.1 Hz, 1H), 4.26 (s, 2H), 3.44 (d, *J* = 12.5 Hz, 2H), 3.31 (s, 1H), 2.98 (t, *J* = 12.9 Hz, 2H), 1.97 (d, *J* = 15.4 Hz, 3H), 1.55 (t, *J* = 11.8 Hz, 2H), 1.26 (s, 1H).

$^{13}$C NMR

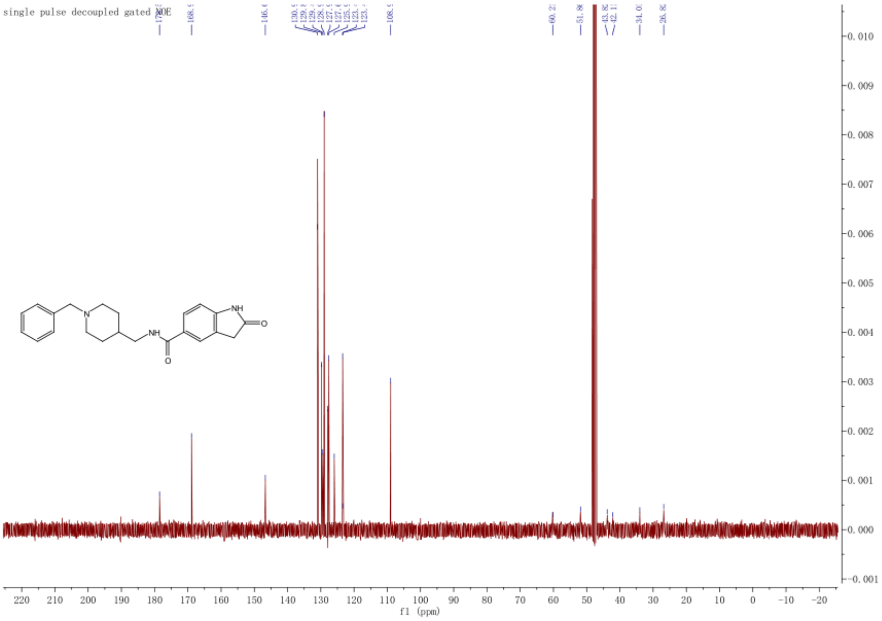

$^{13}$C NMR (101 MHz, $CD_3OD$) δ 178.52, 168.90, 146.69, 130.99, 129.80, 129.40, 128.99, 127.92, 127.60, 125.95, 123.42, 123.40, 108.97, 60.21, 51.86, 43.82, 42.11, 34.01, 26.82.

HRMS (ESI)

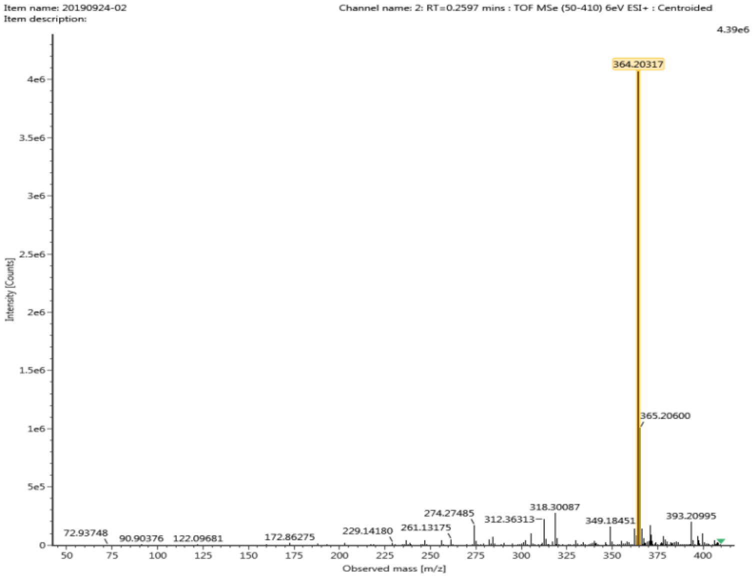


**8j**

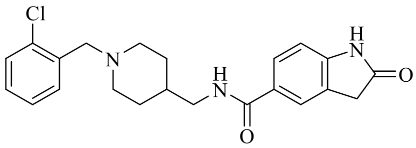

$^{1}$H NMR

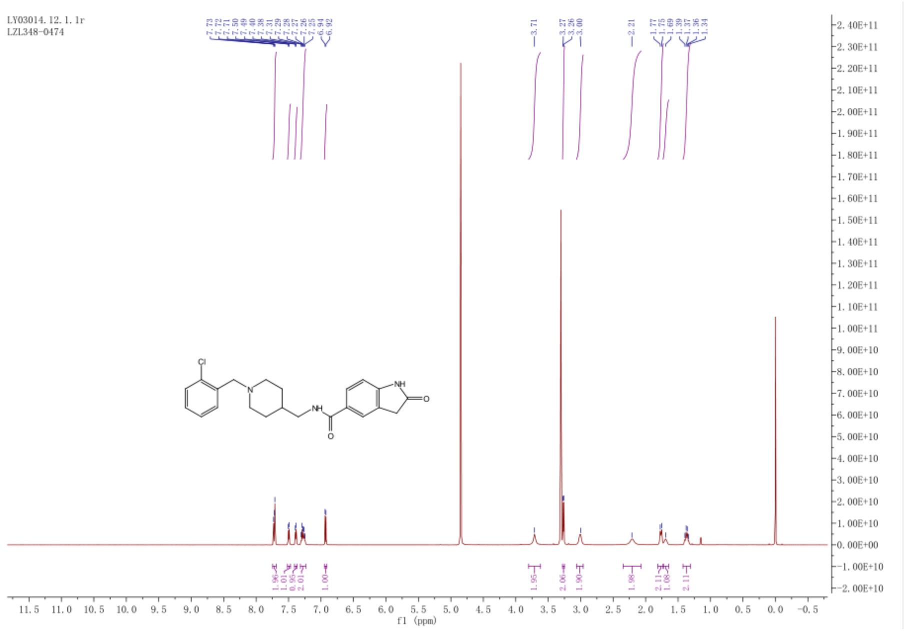


$^{1}$H NMR (600 MHz, $CD_3OD$) δ 7.72 (dd, *J* = 10.5, 2.4 Hz, 2H), 7.50 (d, *J* = 7.2 Hz, 1H), 7.39 (d, *J* = 7.6 Hz, 1H), 7.28 (dt, *J* = 20.1, 7.1 Hz, 2H), 6.93 (d, *J* = 8.1 Hz, 1H), 3.71 (s, 2H), 3.27 (d, *J* = 6.8 Hz, 2H), 3.00 (s, 2H), 2.21 (s, 2H), 1.76 (d, *J* = 12.7 Hz, 2H), 1.69 (s, 1H), 1.37 (q, *J* = 11.5 Hz, 2H).

$^{13}$C NMR

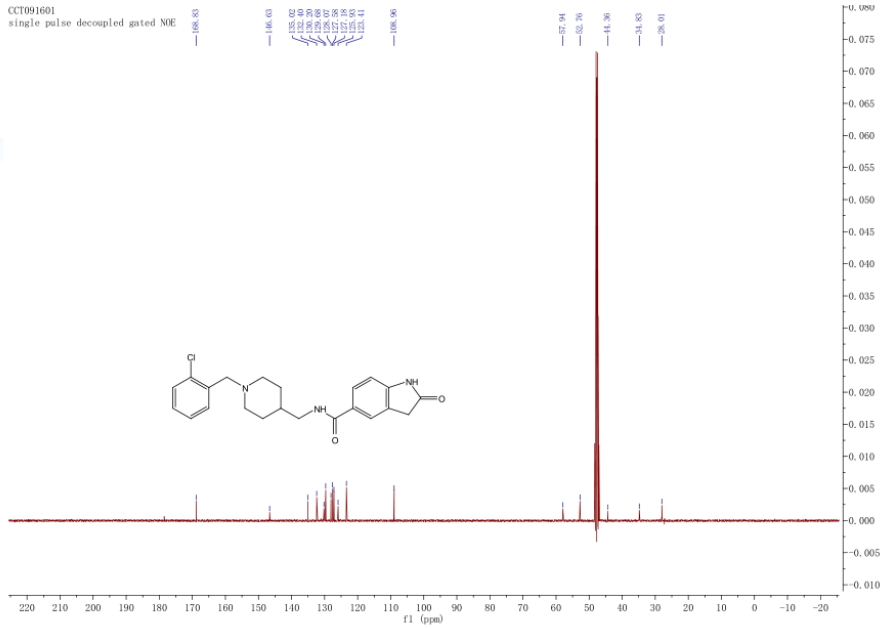


$^{13}C$ NMR (101 MHz, $CD_3OD$) δ 168.83, 146.63, 135.02, 132.40, 130.20, 129.68, 128.07, 127.58, 127.18, 125.93, 123.41, 108.96, 57.94, 52.76, 44.36, 34.83, 28.01.

HRMS (ESI)

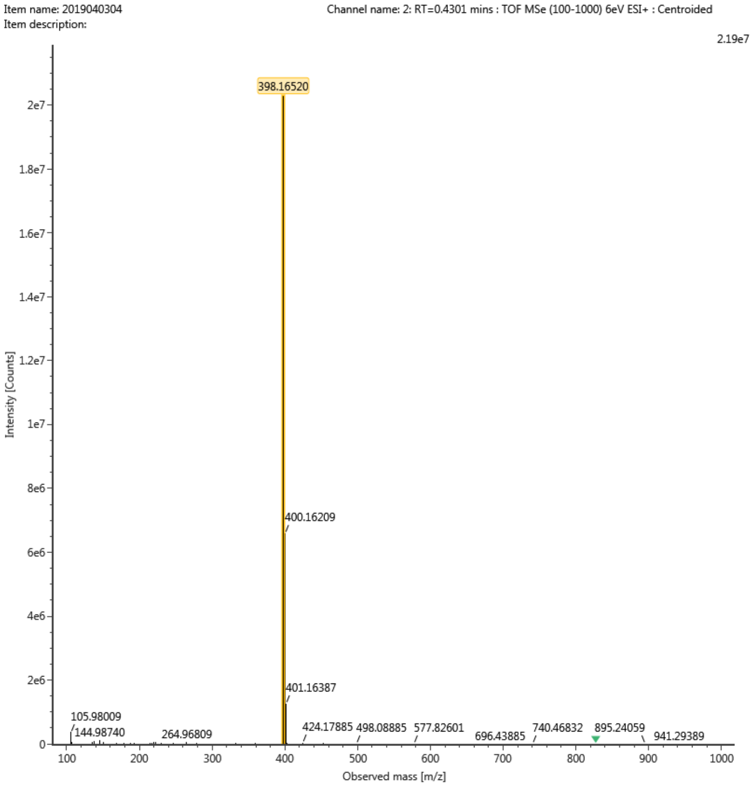


**8k**

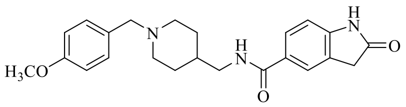


$^{1}H$ NMR

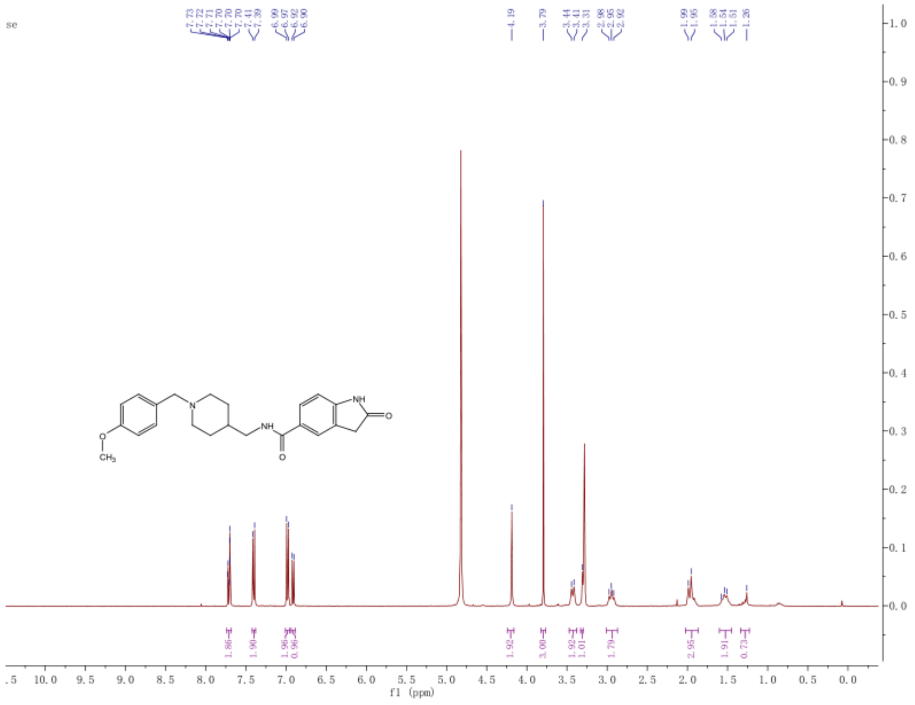

$^{1}$H NMR (400 MHz, $CD_3OD$) δ 7.74 – 7.68 (m, 2H), 7.40 (d, *J* = 8.7 Hz, 2H), 6.98 (d, *J* = 8.7 Hz, 2H), 6.91 (d, *J* = 8.0 Hz, 1H), 4.19 (s, 2H), 3.79 (s, 3H), 3.43 (d, *J* = 12.0 Hz, 2H), 3.31 (s, 1H), 2.95 (t, *J* = 11.7 Hz, 2H), 1.97 (d, *J* = 13.9 Hz, 3H), 1.60 – 1.45 (m, 2H), 1.26 (s, 1H).

$^{13}$C NMR

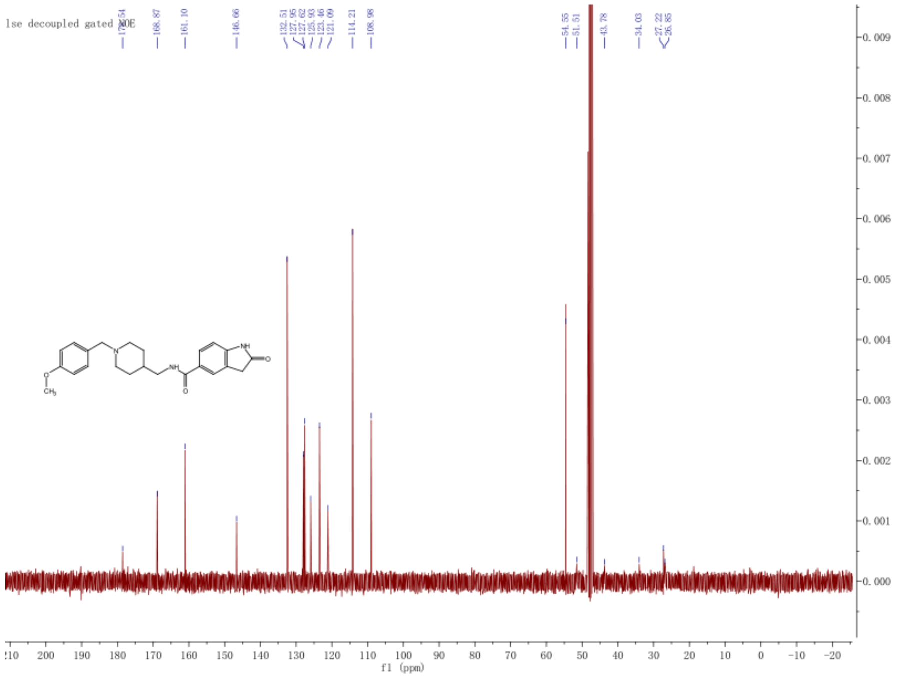

$^{13}$C NMR (101 MHz, $CD_3OD$) δ 178.54, 168.87, 161.10, 146.66, 132.51, 127.95, 127.62, 125.93, 123.46, 121.09, 114.21, 108.98, 54.55, 51.51, 43.78, 34.03, 27.22, 26.85.

HRMS (ESI)

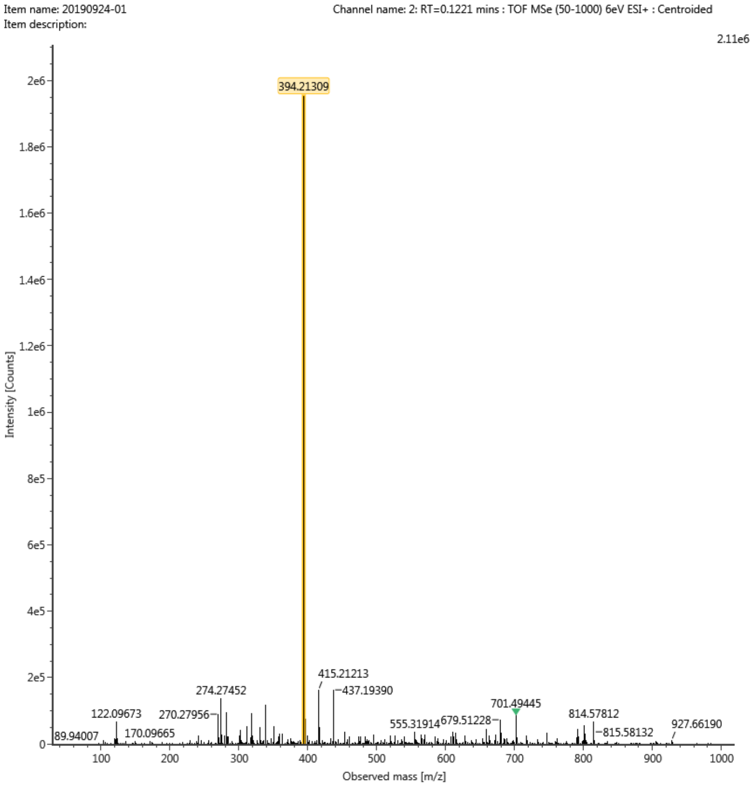


**8l**

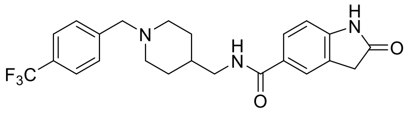

$^1$H NMR

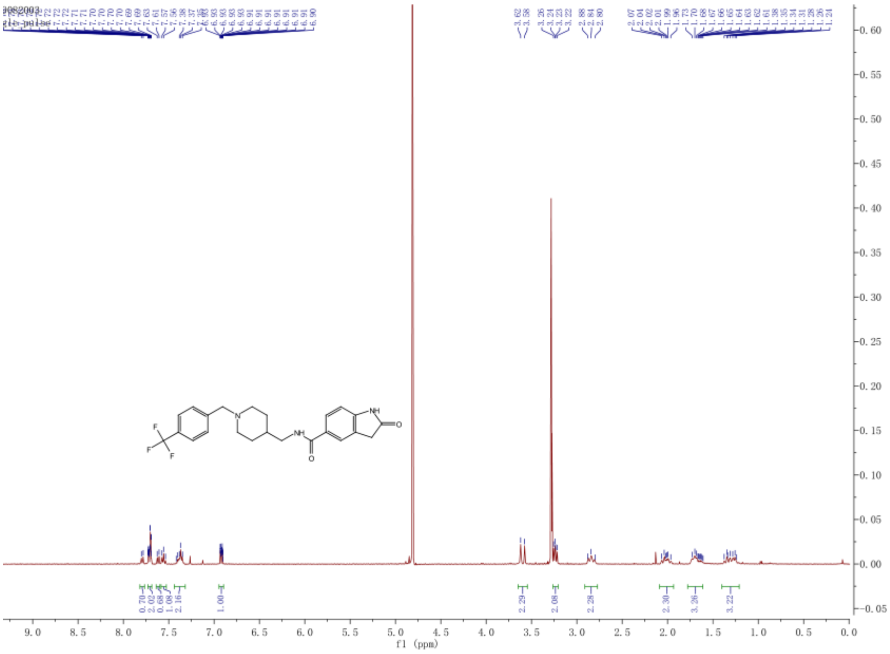


$^1$H NMR (400 MHz, $CD_3OD$) δ 7.79 (d, *J* = 7.8 Hz, 1H), 7.73 – 7.69 (m, 2H), 7.62 (d, *J* = 8.2 Hz, 1H), 7.56 (t, *J* = 7.8 Hz, 1H), 7.44 – 7.32 (m, 2H), 6.95 – 6.90 (m, 1H),

3.60 (d, $J$ = 17.7 Hz, 2H), 3.24 (dd, $J$ = 9.1, 6.8 Hz, 2H), 2.91 – 2.77 (m, 2H), 2.09 – 1.93 (m, 2H), 1.78 – 1.61 (m, 3H), 1.40 – 1.21 (m, 3H).

$^{13}$C NMR

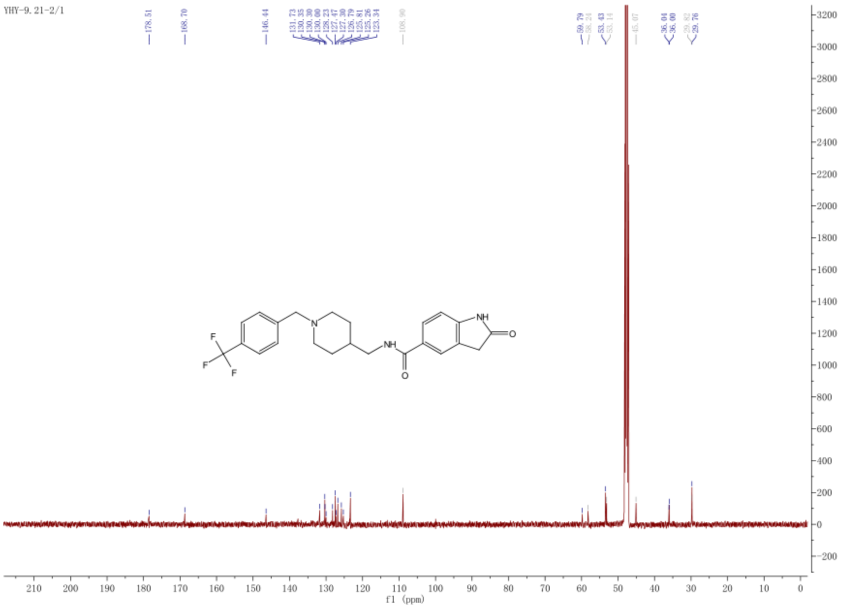

$^{13}$C NMR (126 MHz, $CD_3OD$)) δ 168.70 , 146.44 , 131.73 , 130.35 , 130.30 , 128.23 , 127.47 , 127.30 , 126.79 , 125.81 , 125.26 , 123.34 , 108.90 , 59.79 , 59.79 , 58.24 , 53.43 , 53.14 , 45.07 , 36.04 , 36.04 , 36.00 , 36.00 , 29.82 , 29.76 .

HRMS (ESI)

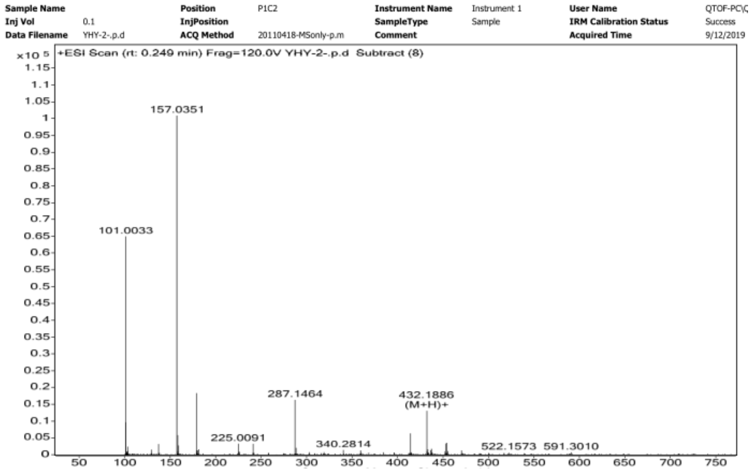


**10s**

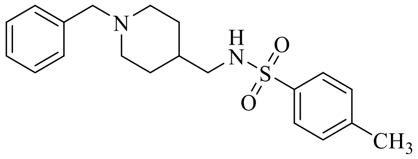

$^{1}$H NMR

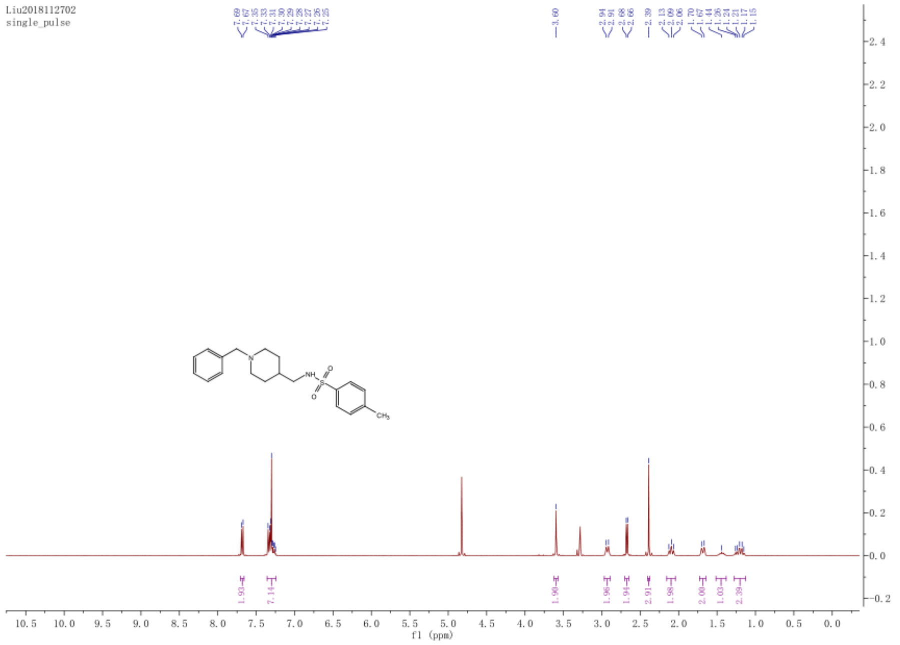


$^{1}$H NMR(400 MHz, $CD_3OD$) δ 7.68 (d, *J* = 8.3 Hz, 2H), 7.36 – 7.24 (m, 7H), 3.60 (s, 2H), 2.93 (d, *J* = 12.0 Hz, 2H), 2.67 (d, *J* = 6.8 Hz, 2H), 2.39 (s, 3H), 2.09 (t, *J* = 12.6 Hz, 2H), 1.68 (d, *J* = 13.8 Hz, 2H), 1.44 (s, 1H), 1.28 – 1.13 (m, 2H).

$^{13}$C NMR

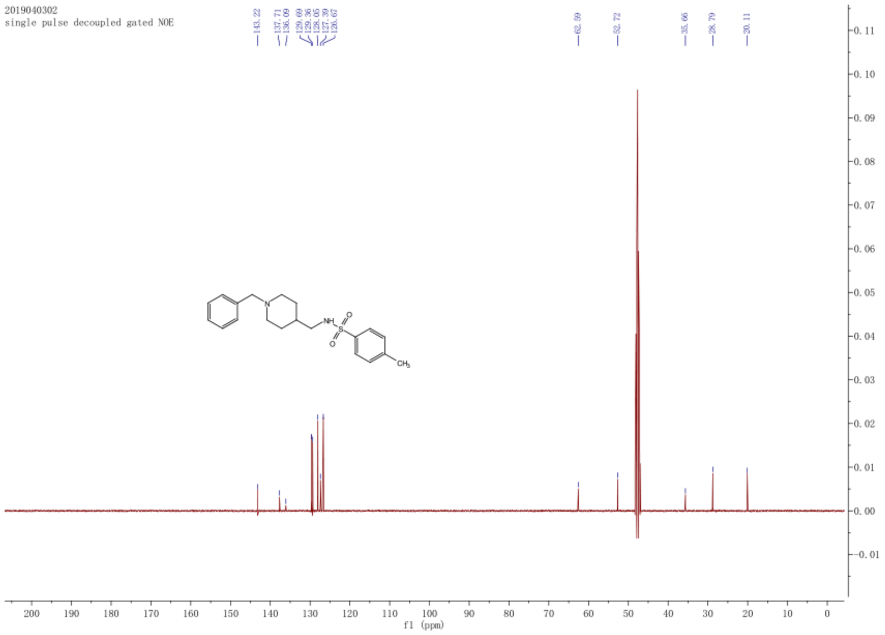


$^{13}$C NMR (101 MHz, $CD_3OD$) δ 143.22, 137.71, 136.09, 129.69, 129.36, 128.05, 127.39, 126.67, 62.59, 52.72, 35.66, 28.79, 20.11.

HRMS (ESI)

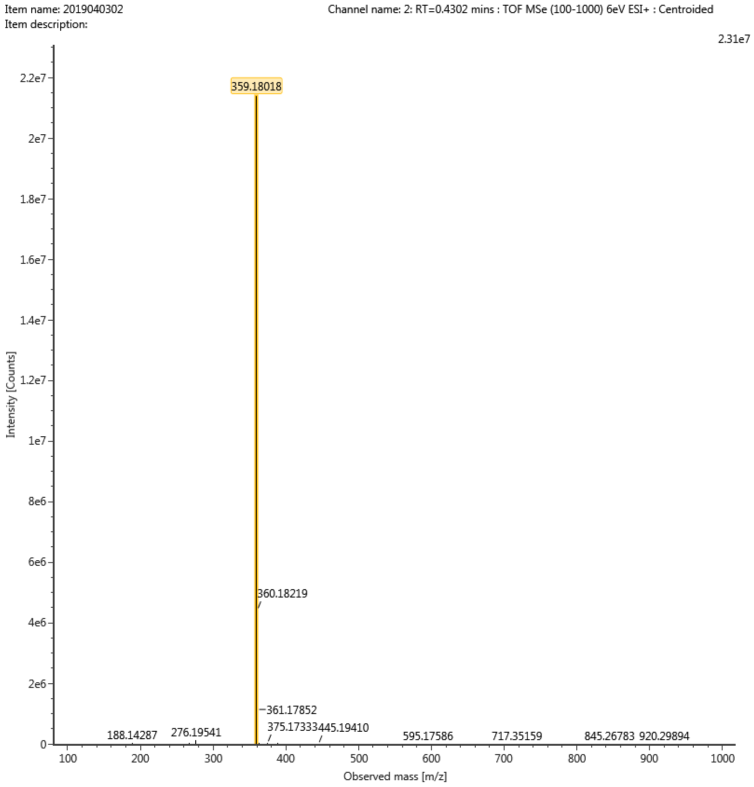


**10t**

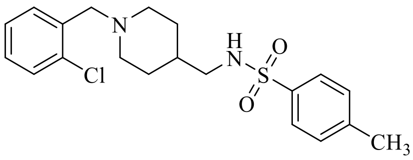


$^1$H NMR

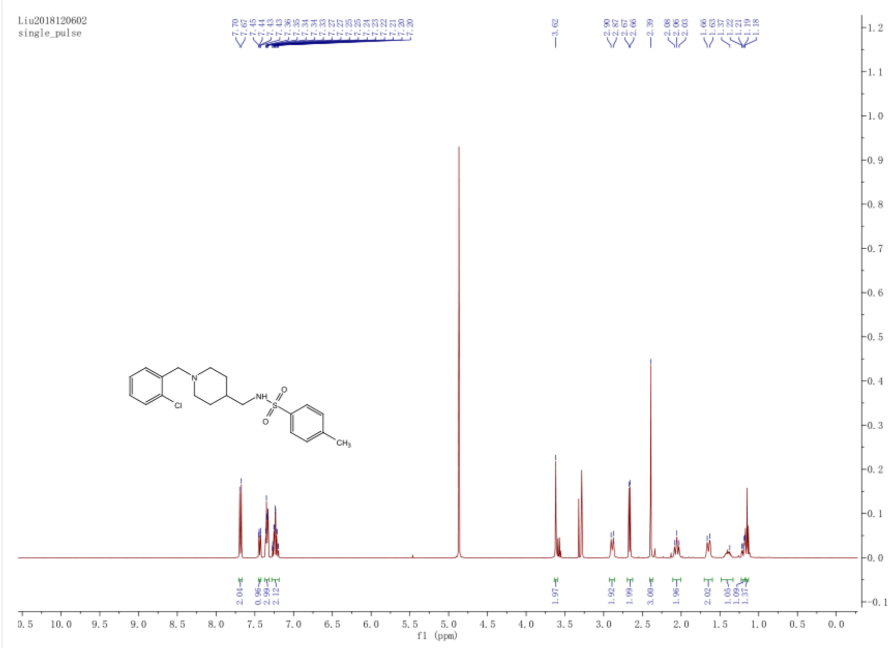


$^1$H NMR (400 MHz, $CD_3OD$) δ 7.69 (d, *J* = 8.3 Hz, 2H), 7.44 (dd, *J* = 7.4, 2.0 Hz, 1H), 7.37 – 7.31 (m, 3H), 7.23 (td, *J* = 7.1, 1.9 Hz, 2H), 3.62 (s, 2H), 2.89 (d, *J* = 11.8

Hz, 2H), 2.67 (d, *J* = 6.8 Hz, 2H), 2.39 (s, 3H), 2.06 (t, *J* = 11.7 Hz, 2H), 1.65 (d, *J* = 13.1 Hz, 2H), 1.37 (s, 1H), 1.20 (dd, *J* = 12.3, 3.6 Hz, 1H), 1.15 (s, 1H).

$^{13}$C NMR

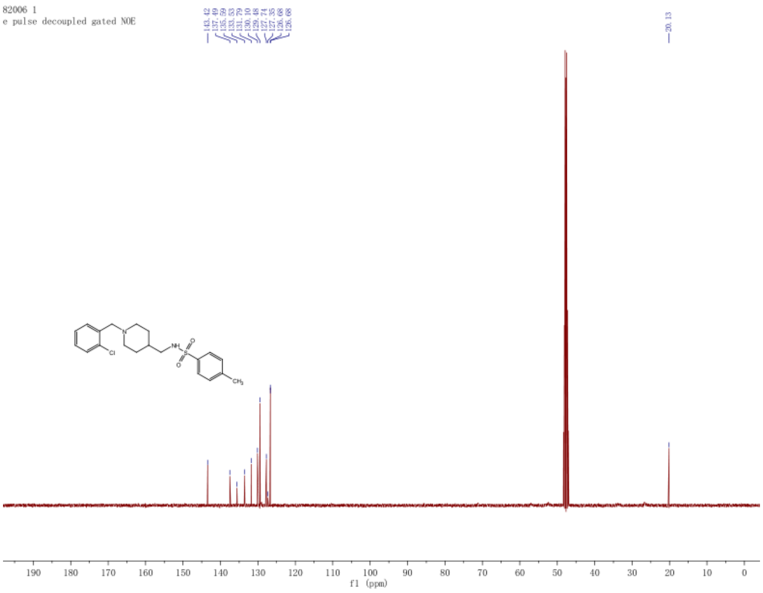


$^{13}$C NMR (101 MHz, $CD_3OD$) δ 143.42, 137.49, 135.59, 133.53, 131.79, 130.10, 129.48, 127.74, 127.35, 126.68, 126.68, 20.13.

HRMS (ESI)

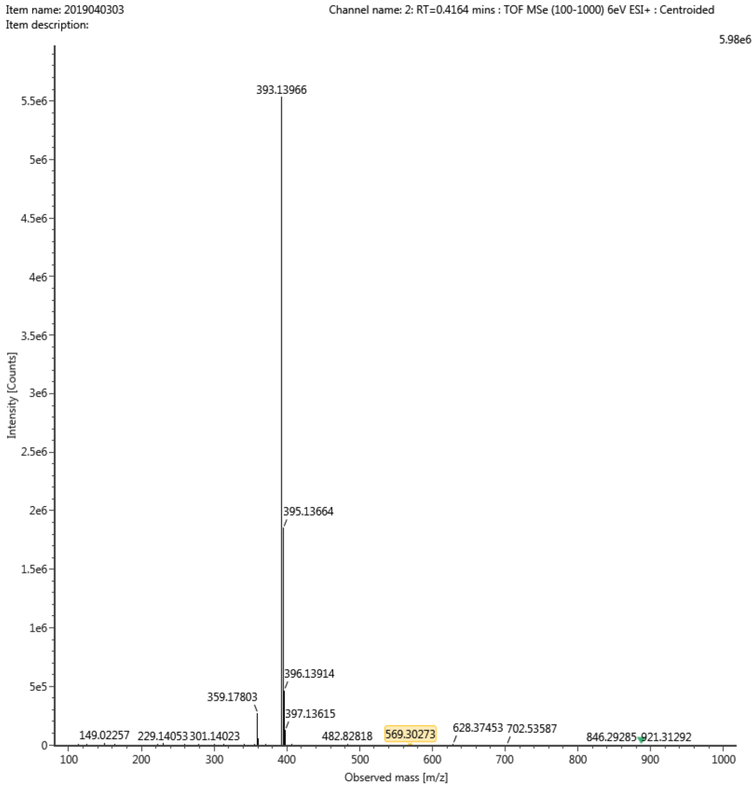


**10u**

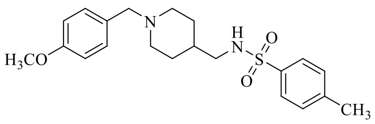

$^{1}$H NMR

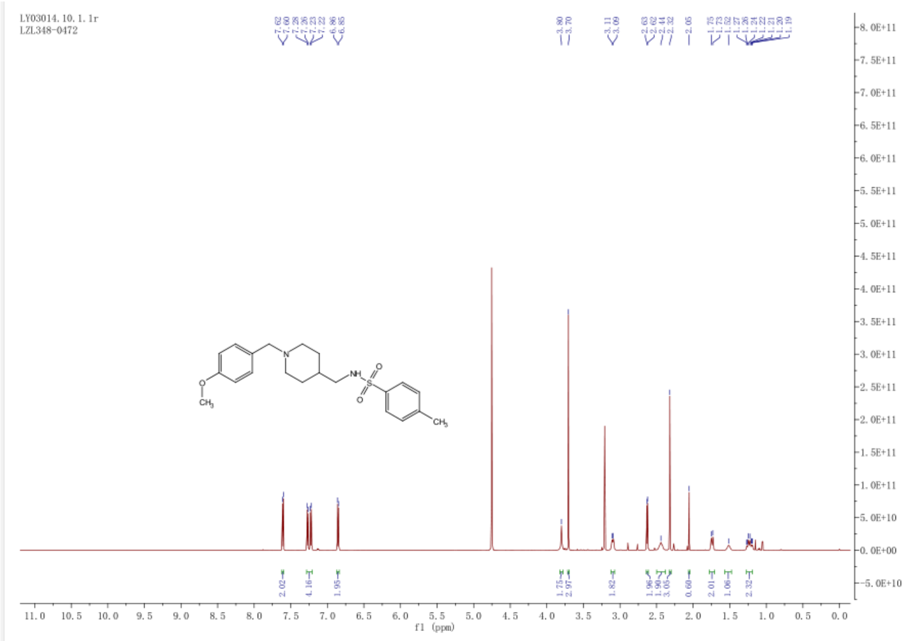

$^{1}$H NMR (600 MHz, $CD_3OD$) δ 7.61 (d, $J$ = 8.3 Hz, 2H), 7.25 (dd, $J$ = 27.4, 8.3 Hz, 4H), 6.85 (d, $J$ = 8.6 Hz, 2H), 3.80 (s, 2H), 3.70 (s, 3H), 3.10 (d, $J$ = 10.7 Hz, 2H), 2.63 (d, $J$ = 6.7 Hz, 2H), 2.44 (s, 2H), 2.32 (s, 3H), 2.05 (s, 1H), 1.73 (s, 2H), 1.52 (s, 1H), 1.27 – 1.19 (m, 2H).

$^{13}$C NMR

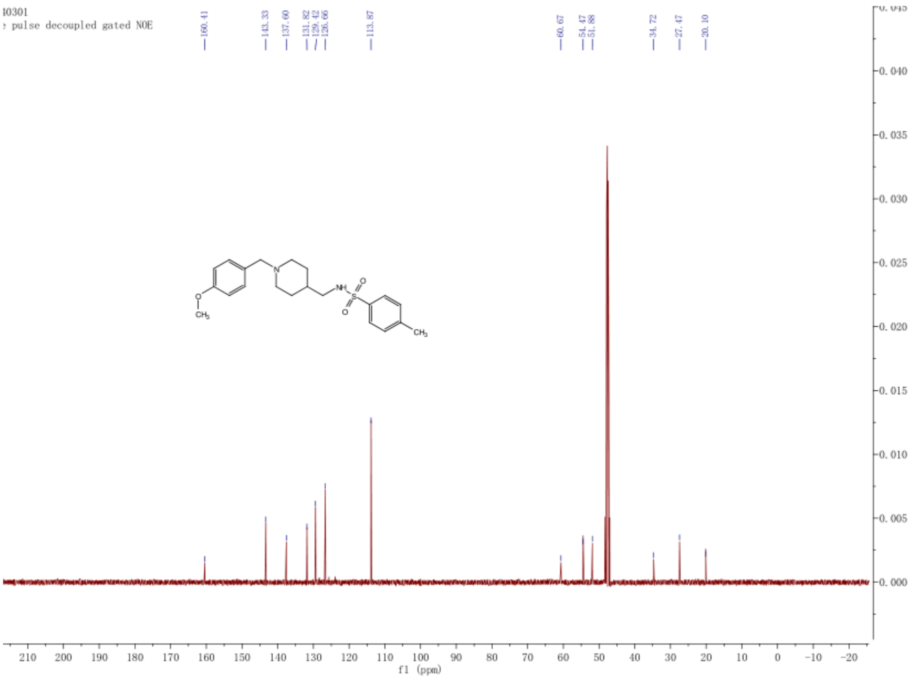

$^{13}$C NMR (101 MHz, $CD_3OD$) δ 160.41, 143.33, 137.60, 131.82, 129.42, 126.66, 113.87, 60.67, 54.47, 51.88, 34.72, 27.47, 20.10.

HRMS (ESI)

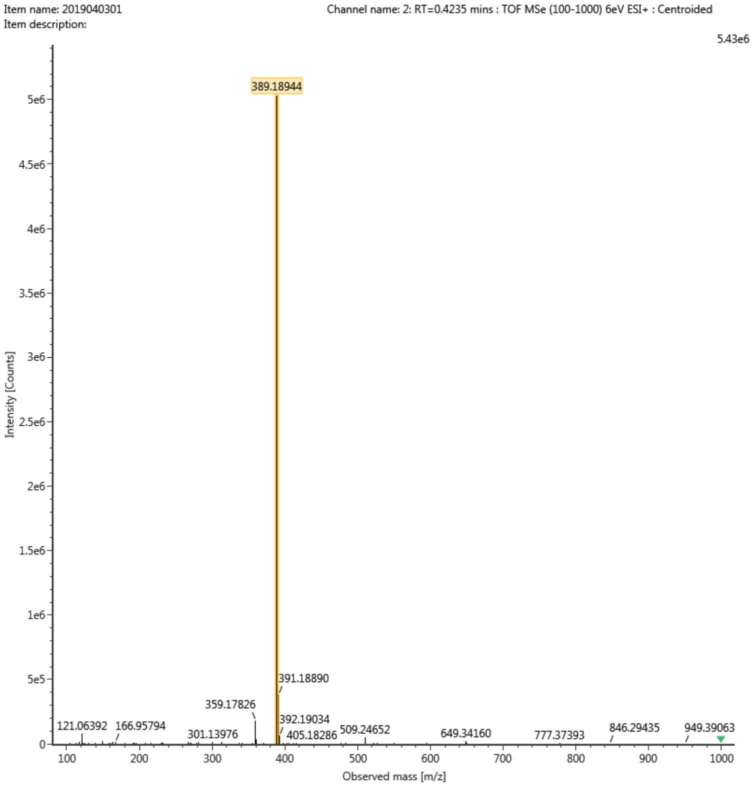


**10v**

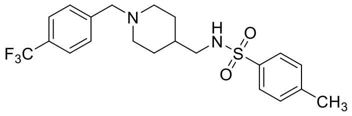


$^{1}$H NMR

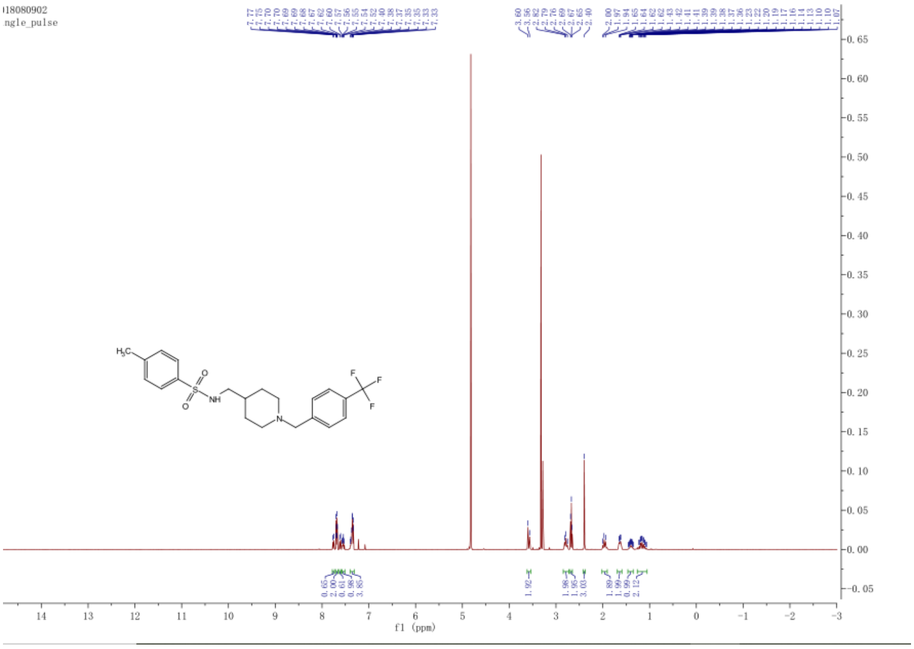


$^{1}$H NMR (400 MHz, $CD_3OD$) δ 7.76 (d, *J* = 7.8 Hz, 1H), 7.71 – 7.66 (m, 2H), 7.61 (d, *J* = 7.9 Hz, 1H), 7.57 – 7.51 (m, 1H), 7.40 – 7.31 (m, 4H), 3.58 (d, *J* = 14.0 Hz, 2H), 2.79 (t, *J* = 12.2 Hz, 2H), 2.67 (t, *J* = 6.9 Hz, 2H), 2.40 (s, 3H), 1.97 (t, *J* = 11.6 Hz,

2H), 1.69 – 1.59 (m, 2H), 1.40 (dtt, *J* = 14.5, 7.3, 3.2 Hz, 1H), 1.15 (dqd, *J* = 24.7, 12.2, 3.7 Hz, 2H).

$^{13}$C NMR

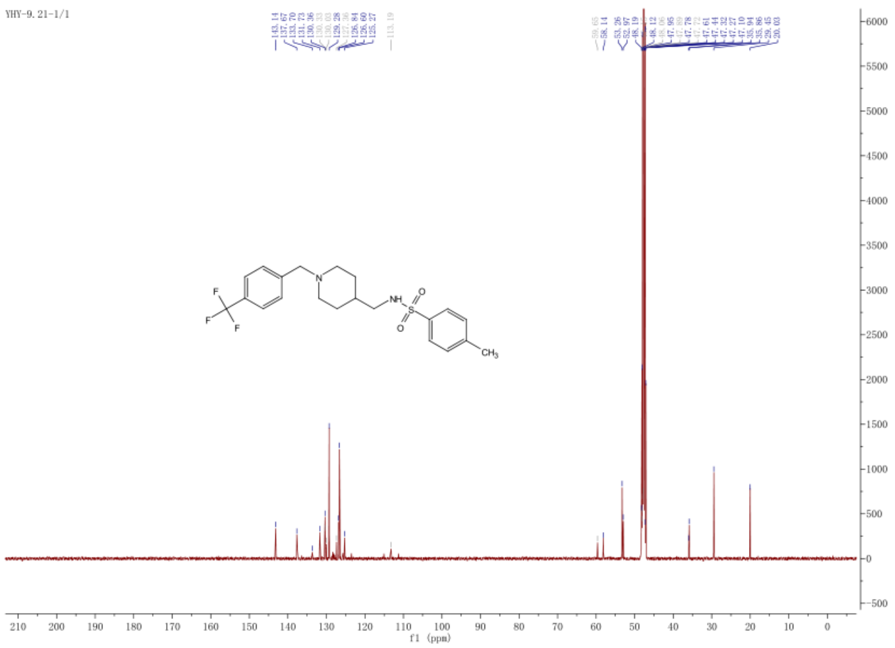


$^{13}$C NMR (126 MHz, $CD_3OD$) δ 143.14 , 137.67 , 131.73 , 130.36 , 130.33 , 130.03 , 129.28 , 127.36 , 126.84 , 126.60 , 125.27 , 59.65 , 58.14 , 53.26 , 52.97 , 35.94 , 35.86 , 29.45 , 20.03.

HRMS (ESI)

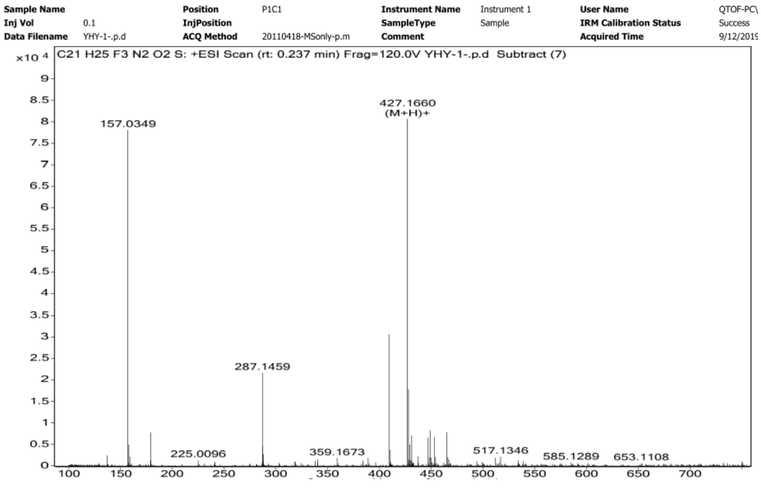


**16a**

$^{1}$H NMR

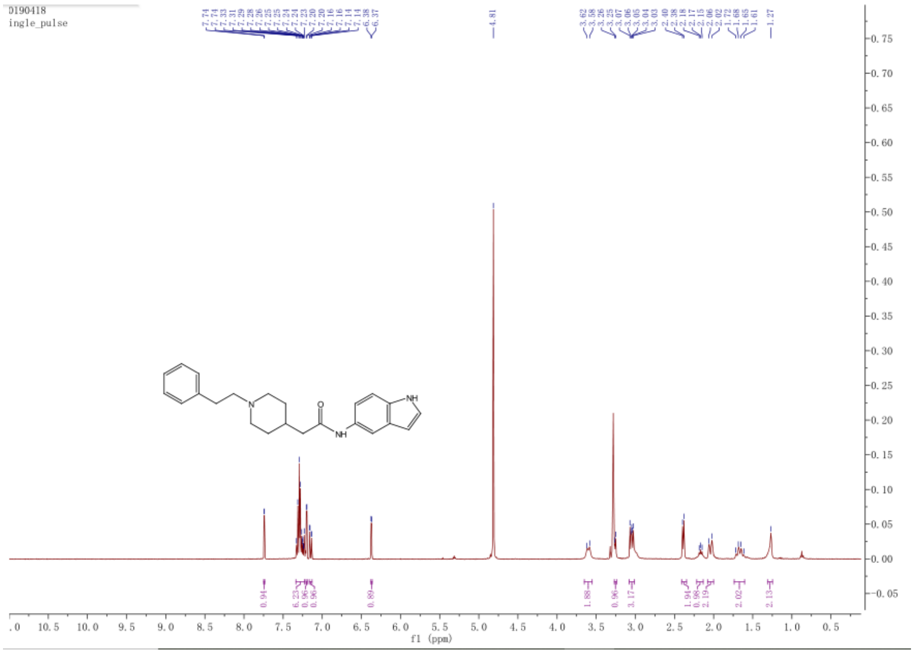

$^1H$ NMR (400 MHz, $CD_3OD$) δ 7.74 (d, *J* = 2.0 Hz, 1H), 7.28 (tt, *J* = 13.1, 6.8 Hz, 6H), 7.20 (d, *J* = 3.1 Hz, 1H), 7.15 (dd, *J* = 8.6, 2.0 Hz, 1H), 6.37 (d, *J* = 3.1 Hz, 1H), 3.60 (d, *J* = 14.9 Hz, 2H), 3.26 (d, *J* = 5.0 Hz, 1H), 3.08 – 3.01 (m, 3H), 2.39 (d, *J* = 7.0 Hz, 2H), 2.21 – 2.13 (m, 1H), 2.04 (d, *J* = 15.4 Hz, 2H), 1.74 – 1.60 (m, 2H), 1.27 (s, 2H).

$^{13}C$ NMR

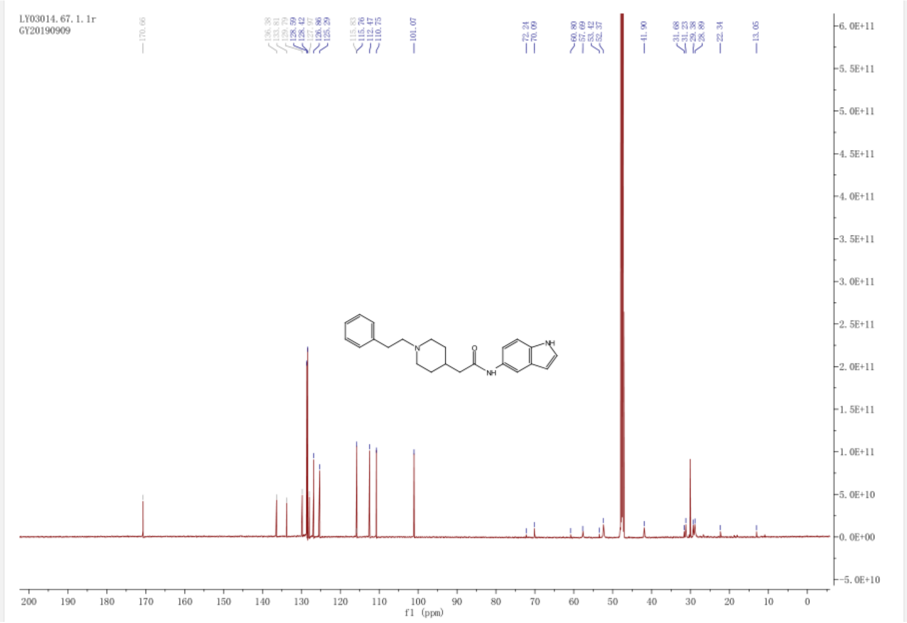

$^{13}C$ NMR (151 MHz, $CD_3OD$) δ 170.66, 136.38, 133.81, 129.79, 128.59, 128.42, 127.97, 126.86, 125.29, 115.83, 115.76, 112.47, 110.75, 101.07, 72.24, 70.09, 60.80, 57.69, 53.42, 52.37, 41.90, 31.68, 31.23, 29.38, 28.89, 22.34, 13.05.

HRMS (ESI)

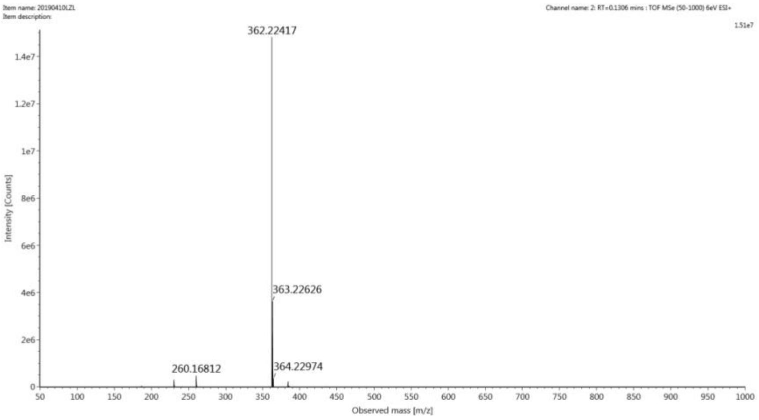


**16b**

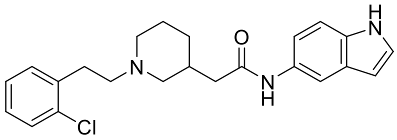


$^1$H NMR

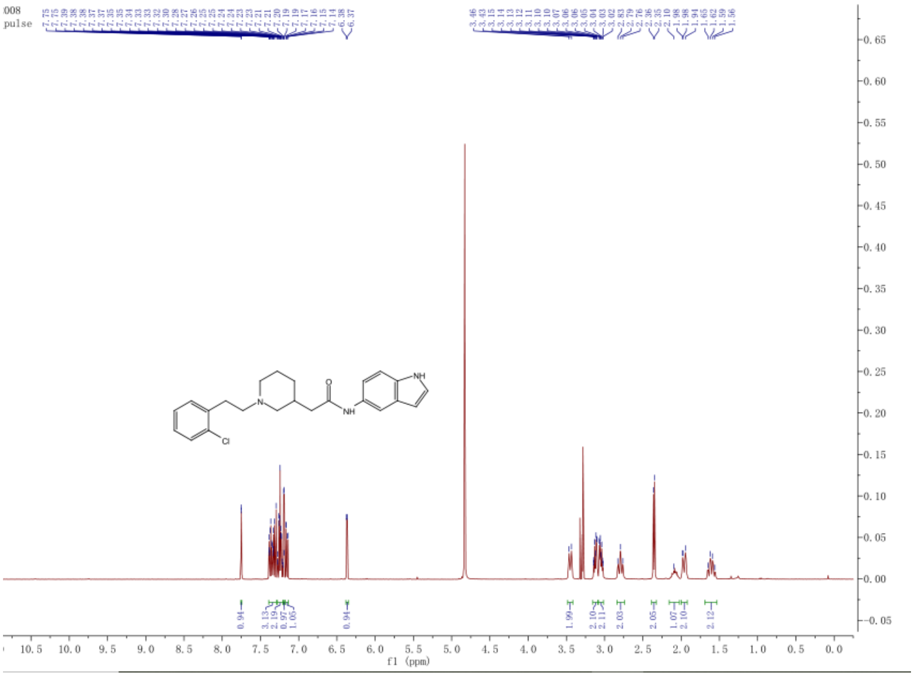


$^1$H NMR (400 MHz, $CD_3OD$) δ 7.75 (d, *J* = 1.9 Hz, 1H), 7.39 – 7.29 (m, 3H), 7.28 – 7.20 (m, 2H), 7.19 (d, *J* = 3.1 Hz, 1H), 7.16 (dd, *J* = 8.7, 2.0 Hz, 1H), 6.37 (d, *J* = 4.0 Hz, 1H), 3.45 (d, *J* = 12.3 Hz, 2H), 3.15 – 3.09 (m, 2H), 3.08 – 3.01 (m, 2H), 2.79 (t, *J* = 13.0 Hz, 2H), 2.36 (d, *J* = 7.1 Hz, 2H), 2.10 (s, 1H), 2.00 – 1.92 (m, 2H), 1.60 (q, *J* = 11.5 Hz, 2H).

$^{13}$C NMR

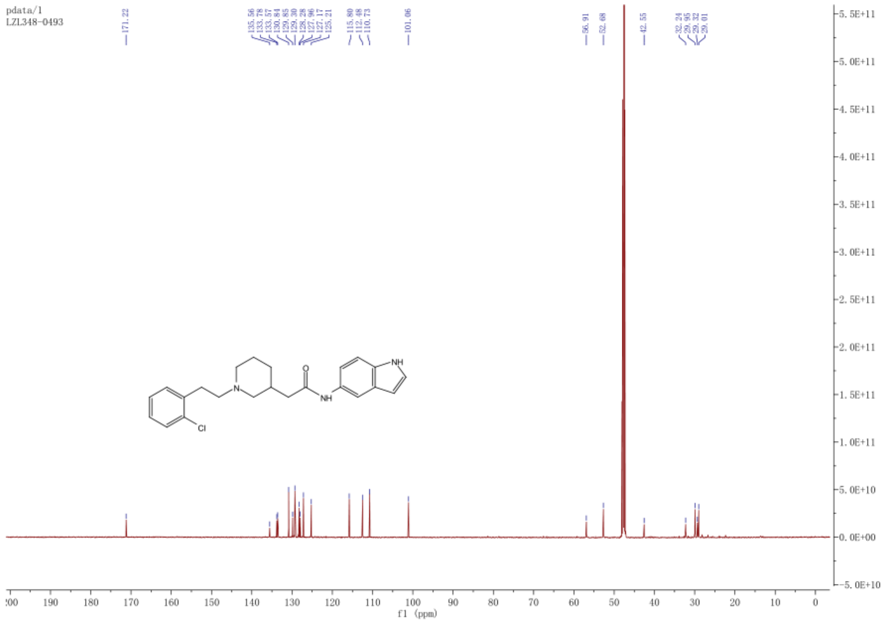

$^{13}$C NMR (151 MHz, $CD_3OD$) δ 171.22, 135.56, 133.78, 133.57, 130.84, 129.85, 129.30, 128.28, 127.96, 127.17, 125.21, 115.80, 112.48, 110.73, 101.06, 56.91, 52.68, 42.55, 32.24, 29.95, 29.32, 29.01.

HRMS (ESI)

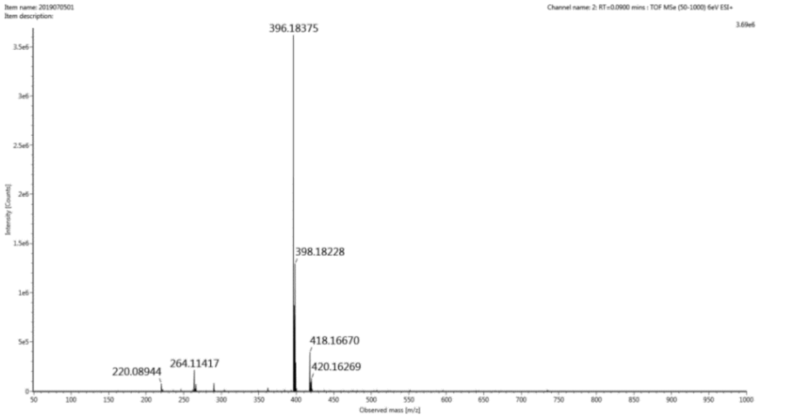


**16c**

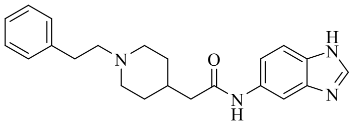

$^{1}$H NMR

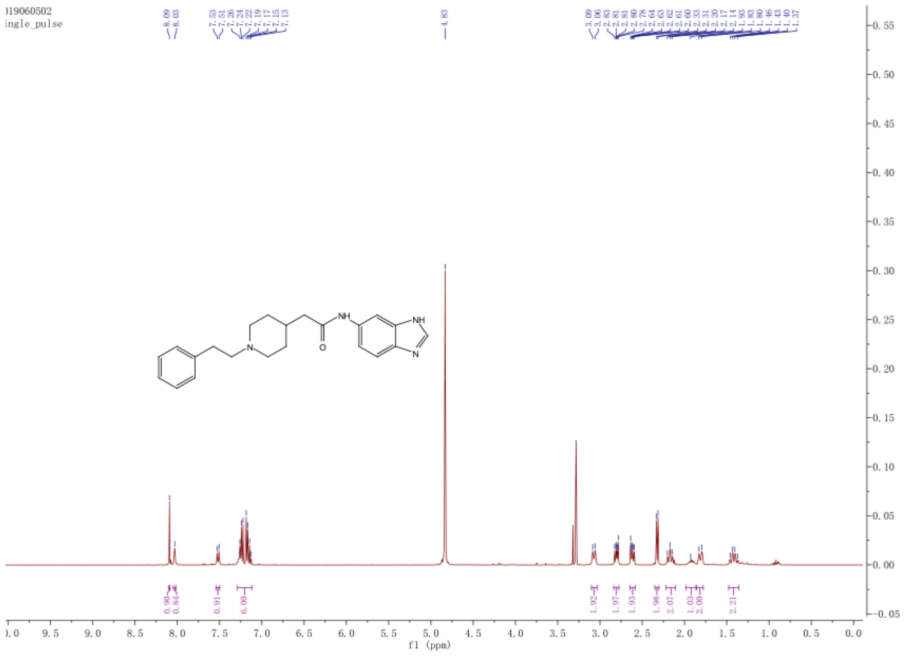

$^{1}H$ NMR (400 MHz, $CD_3OD$) δ 8.09 (s, 1H), 8.03 (s, 1H), 7.52 (d, *J* = 8.7 Hz, 1H), 7.29 – 7.12 (m, 6H), 3.07 (d, *J* = 12.0 Hz, 2H), 2.84 – 2.78 (m, 2H), 2.64 – 2.58 (m, 2H), 2.32 (d, *J* = 7.2 Hz, 2H), 2.17 (t, *J* = 11.8 Hz, 2H), 1.93 (s, 1H), 1.81 (d, *J* = 12.9 Hz, 2H), 1.42 (q, *J* = 12.1, 10.5 Hz, 2H).

$^{13}C$ NMR

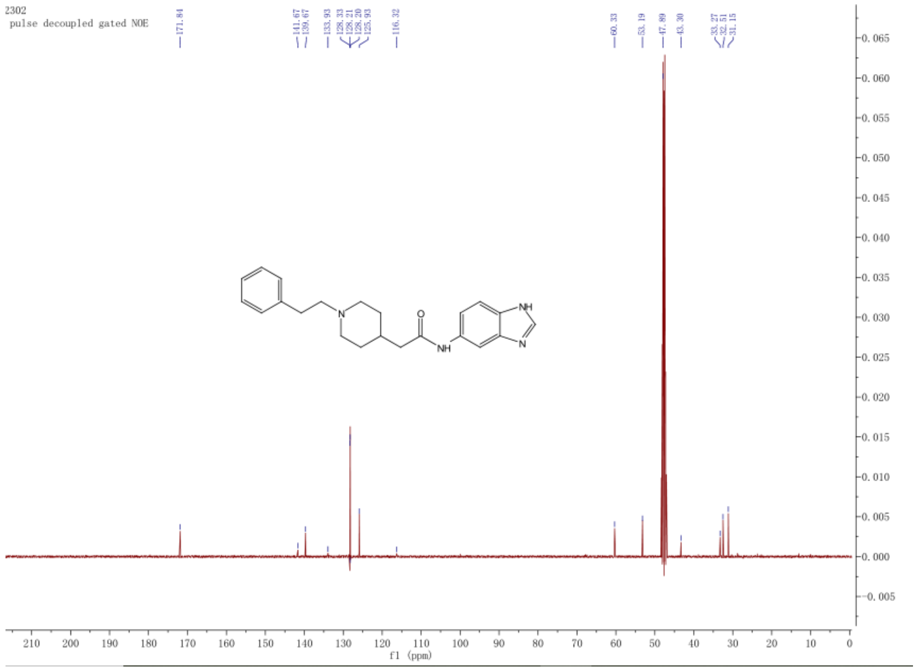

$^{13}C$ NMR (101 MHz, $CD_3OD$) δ 171.84, 141.67, 139.67, 133.93, 128.33, 128.21, 128.20, 125.93, 116.32, 60.33, 53.19, 47.89, 43.30, 33.27, 32.51, 31.15.

HRMS (ESI)

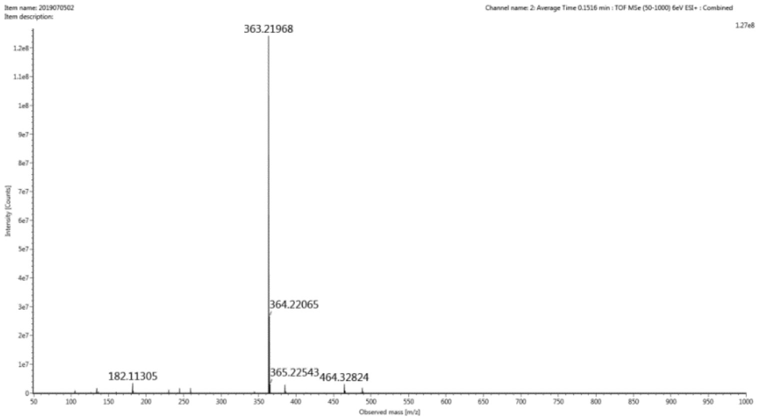


**16d**

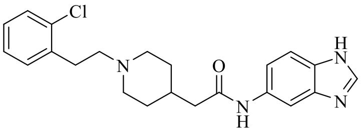


$^{1}$H NMR

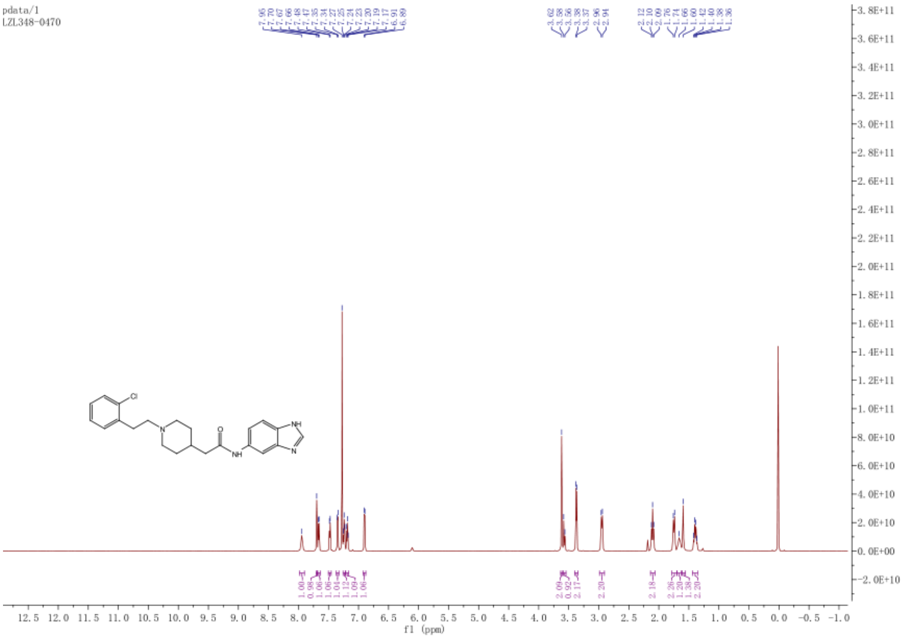


$^{1}$H NMR (600 MHz, $CDCl_3$) δ 7.95 (s, 1H), 7.70 (s, 1H), 7.66 (d, *J* = 8.1 Hz, 1H), 7.48 (d, *J* = 7.4 Hz, 1H), 7.35 (d, *J* = 7.8 Hz, 1H), 7.24 (t, *J* = 7.4 Hz, 1H), 7.19 (t, *J* = 7.3 Hz, 1H), 6.90 (d, *J* = 8.1 Hz, 1H), 3.62 (s, 2H), 3.57 (d, *J* = 11.7 Hz, 1H), 3.37 (d, *J* = 6.5 Hz, 2H), 2.95 (d, *J* = 11.2 Hz, 2H), 2.10 (t, *J* = 11.3 Hz, 2H), 1.75 (d, *J* = 12.4 Hz, 2H), 1.66 (s, 1H), 1.60 (s, 1H), 1.39 (q, *J* = 11.5 Hz, 2H).

$^{13}$C NMR

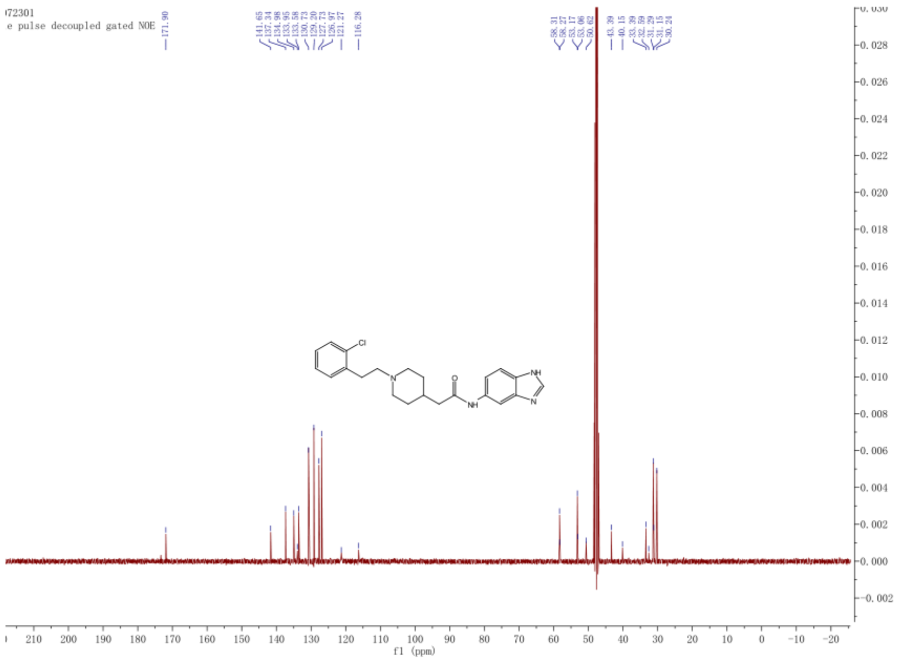


$^{13}$C NMR (101 MHz, $CD_3OD$) δ 171.90, 141.65, 137.34, 134.98, 133.95, 133.58, 130.73, 129.20, 127.73, 126.97, 121.27, 116.28, 58.31, 58.27, 53.17, 53.06, 50.62, 43.39, 40.15, 33.39, 32.59, 31.29, 31.15, 30.24.

HRMS (ESI)

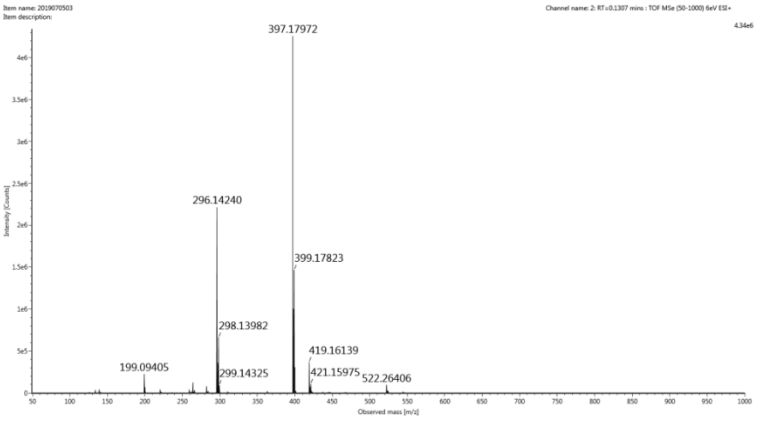

Supplement: Supplementary file 1 [file molecules-25-00489-s001.pdf]
